# Supplementary material for: Changes in infant head shape: Developmental trends during the first year of life and secular changes observed in recent years
Source: PLoS One. 2026 Mar 13;21(3):e0344700. doi: 10.1371/journal.pone.0344700 (PMC12987498; doi:10.1371/journal.pone.0344700)
Supplement: S1 File — (PDF) [file pone.0344700.s001.pdf]

Supplementary Tables

Title

Changes in Infant Head Shape: Developmental Trends During the First Year of Life and Secular Changes Observed in Recent Years

Short title

Developmental and Secular Changes in the Infant Head Shape

Eujin Lee[1], Hama Watanabe[1], Ryoya Saji[2,3], Fumitaka Homae[4,5] Gentaro Taga[1]

[1] Graduate School of Education, The University of Tokyo, Tokyo, Japan

[2] College of Agriculture, Tamagawa University, Tokyo, Japan

[3] Brain Science Institute, Tamagawa University, Tokyo, Japan

[4] Department of Language Sciences, Tokyo Metropolitan University, Tokyo, Japan

[5] Research Center for Language, Brain and Genetics, Tokyo Metropolitan University, Tokyo, Japan

Corresponding author: Eujin Lee

Email: elee715@p.u-tokyo.ac.jp

S1 Table. ANOVA tables for direct measurements.

a) Left Tragion-Right Tragion (LT-RT)

	Sum Sq	F value	P value	Significance
Age (months)	1011.87	215.55	< 0.001	***
Birth year	104.64	7.43	< 0.001	***
Sex	159.41	101.88	< 0.001	***
Residuals	1400.47			

b) Glabella-Occipital protuberance (G-O)

	Sum Sq	F value	P value	Significance
Age (months)	384.60	90.18	< 0.001	***
Birth year	51.79	4.05	< 0.001	***
Sex	48.67	34.23	< 0.001	***
Residuals	1272.37			

c) Head Circumference (HC)

	Sum Sq	F value	P value	Significance
Age (months)	781.22	248.05	< 0.001	***
Birth year	21.63	2.29	0.02	*
Sex	174.97	166.67	< 0.001	***
Residuals	939.58			

Sum Sq: Sum of squares.

For all tables, df = 3 for age (months), df = 9 for birth year, df = 1 for sex, df = 895 for residuals.

For all tables, *: p < 0.05, **: p < 0.01, and ***: p < 0.001.

S2 Table. Estimated marginal means (EMM), pairwise mean differences, and effect sizes for Left Tragion-Right Tragion (LT-RT) across age groups.

a) Estimated marginal means (EMM)

	EMM	SE	Lower CL	Upper CL
2 months	27.38	0.12	27.15	27.62
3 months	28.55	0.06	28.43	28.67
6 months	31.53	0.16	31.22	31.84
12 months	32.88	0.26	32.36	33.40

SE: Standard error of the EMM. CL: 95% Confidence limit. df = 895.

b) EMM difference

Contrast	EMM difference	SE	t ratio	P value	Significance
2 months - 3 months	-1.17	0.14	-8.33	< 0.001	***
2 months - 6 months	-4.15	0.21	-19.84	< 0.001	***
2 months - 12 months	-5.49	0.30	-18.43	< 0.001	***
3 months - 6 months	-2.98	0.18	-16.79	< 0.001	***
3 months - 12 months	-4.33	0.27	-16.19	< 0.001	***
6 months - 12 months	-1.35	0.31	-4.30	< 0.001	***

SE: standard error of the estimated difference between groups. df = 895.

c) Effect size

Contrast	Effect size	SE	Lower CL	Upper CL
2 months - 3 months	-0.93	0.11	-1.16	-0.71
2 months - 6 months	-3.31	0.18	-3.68	-2.95
2 months - 12 months	-4.39	0.26	-4.90	-3.88
3 months - 6 months	-2.38	0.15	-2.68	-2.08
3 months - 12 months	-3.46	0.23	-3.91	-3.01
6 months - 12 months	-1.08	0.25	-1.57	-0.58

SE: standard error of the Effect size estimate. CL: Confidence level. df = 895.

S3 Table. Estimated marginal means (EMM), pairwise mean differences, and effect sizes for Glabella-Occipital protuberance (G-O) across age groups.

a) Estimated marginal means (EMM)

Age group	EMM	SE	Lower CL	Upper CL
2 months	23.32	0.11	23.10	23.54
3 months	24.19	0.06	24.07	24.30
6 months	25.20	0.15	24.91	25.50
12 months	27.62	0.25	27.12	28.11

SE: Standard error of the EMM. CL: Confidence limit. df = 895.

b) EMM difference

Contrast	EMM difference	SE	t ratio	P value	Significance
2 months - 3 months	-0.87	0.13	-6.51	< 0.001	***
2 months - 6 months	-1.89	0.20	-9.47	< 0.001	***
2 months - 12 months	-4.30	0.28	-15.13	< 0.001	***
3 months - 6 months	-1.02	0.17	-6.01	< 0.001	***
3 months - 12 months	-3.43	0.25	-13.46	< 0.001	***
6 months - 12 months	-2.41	0.30	-8.07	< 0.001	***

SE: standard error of the estimated difference between groups. df = 895.

c) Effect size

Contrast	Effect size	SE	Lower CL	Upper CL
2 months - 3 months	-0.73	0.11	-0.95	-0.51
2 months - 6 months	-1.58	0.17	-1.92	-1.25
2 months - 12 months	-3.60	0.25	-4.10	-3.11
3 months - 6 months	-0.85	0.14	-1.13	-0.57
3 months - 12 months	-2.87	0.22	-3.31	-2.43
6 months - 12 months	-2.02	0.26	-2.52	-1.52

SE: standard error of the Effect size estimate. CL: Confidence level. df = 895.

S4 Table. Estimated marginal means (EMM), pairwise mean differences, and effect sizes for head circumference (HC) across age groups.

a) Estimated marginal mean (EMM)

	EMM	SE	Lower CL	Upper CL
2 months	40.09	0.10	39.90	40.28
3 months	41.03	0.05	40.93	41.13
6 months	43.21	0.13	42.96	43.47
12 months	45.54	0.22	45.12	45.96

SE: Standard error of the EMM. CL: Confidence limit. df = 895.

b) EMM difference

Contrast	EMM difference	SE	t ratio	P value	Significance
2 months - 3 months	-0.94	0.11	-8.18	< 0.001	***
2 months - 6 months	-3.13	0.17	-18.27	< 0.001	***
2 months - 12 months	-5.45	0.24	-22.33	< 0.001	***
3 months - 6 months	-2.19	0.15	-15.06	< 0.001	***
3 months - 12 months	-4.51	0.22	-20.62	< 0.001	***
6 months - 12 months	-2.32	0.26	-9.06	< 0.001	***

SE: standard error of the estimated difference between groups. df = 895.

c) Effect size

Contrast	Effect size	SE	Lower CL	Upper CL
2 months - 3 months	-0.92	0.11	-1.14	-0.69
2 months - 6 months	-3.05	0.18	-3.41	-2.70
2 months - 12 months	-5.32	0.27	-5.85	-4.79
3 months - 6 months	-2.14	0.15	-2.43	-1.84
3 months - 12 months	-4.40	0.24	-4.87	-3.94
6 months - 12 months	-2.27	0.26	-2.77	-1.77

SE: standard error of the Effect size estimate. CL: Confidence level. df = 895.

S5 Table. ANOVA tables for estimated measurements.

a) Half-width (a_1)

	Sum Sq	F value	P value	Significance
Age (months)	46.36	76.76	< 0.001	***
Birth year	14.13	7.80	< 0.001	***
Sex	9.61	47.73	< 0.001	***
Residuals	180.19			

b) Half-length (a_2)

	Sum Sq	F value	P value	Significance
Age (months)	12.89	20.15	< 0.001	***
Birth year	21.51	11.21	< 0.001	***
Sex	1.23	5.78	0.02	*
Residuals	190.87			

c) Height (h)

	Sum Sq	F value	P value	Significance
Age (months)	93.80	122.06	< 0.001	***
Birth year	9.36	4.06	< 0.001	***
Sex	11.09	43.30	< 0.001	***
Residuals	229.26			

d) Cephalic index (CI)

	Sum Sq	F value	P value	Significance
Age (months)	7574.88	17.97	< 0.001	***
Birth year	12192.16	9.64	< 0.001	***
Sex	1062.53	7.56	0.006	**
Residuals	125768.02			

e) Height-half-length ratio (h/a_2)

	Sum Sq	F value	P value	Significance
Age (months)	1.05	36.90	< 0.001	***
Birth year	1.08	12.64	< 0.001	***
Sex	0.08	8.95	0.003	**
Residuals	8.47			

f) Volume

	Sum Sq	F value	P value	Significance
Age (months)	4369406.86	353.34	< 0.001	***
Birth year	49979.90	1.35	0.21	ns
Sex	633111.79	153.59	< 0.001	***
Residuals	3689229.32			

g) Globularity index (GI)

	Sum Sq	F value	P value	Significance
Age (months)	0.001	4.49	0.004	**
Birth year	0.003	4.25	< 0.001	***
Sex	1.98×10^{-5}	0.23	0.63	ns
Residuals	0.08			

Sum Sq: Sum of squares. For all tables, df = 3 for age (months), df = 9 for birth year, df = 1 for sex, df = 895 for residuals.

For GI, sum of squares are presented to three decimal places, as rounding up to two decimal places produces zero for most entries. Small values are reported in scientific notation with three significant figures.

S6 Table. Estimated marginal means (EMM), mean differences, and Effect sizes for half-width (a_1) across age groups.

a) Estimated marginal mean (EMM)

Age group	EMM	SE	Lower CL	Upper CL
2 months	5.93	0.04	5.85	6.01
3 months	6.10	0.02	6.06	6.14
6 months	6.88	0.06	6.77	6.99
12 months	6.83	0.09	6.64	7.02

SE: Standard error of the EMM. CL: Confidence limit. df = 895.

b) EMM difference

Contrast	EMM difference	SE	t ratio	P value	Significance
2 months - 3 months	-0.17	0.05	-3.38	0.001	***
2 months - 6 months	-0.95	0.07	-12.71	< 0.001	***
2 months - 12 months	-0.90	0.11	-8.41	< 0.001	***
3 months - 6 months	-0.78	0.06	-12.31	< 0.001	***
3 months - 12 months	-0.73	0.10	-7.60	< 0.001	***
6 months - 12 months	0.05	0.11	0.48	0.63	ns

SE: standard error of the estimated difference between groups. df = 895.

c) Effect size

Contrast	Effect size	SE	Lower CL	Upper CL
2 months - 3 months	-0.38	0.11	-0.60	-0.16
2 months - 6 months	-2.12	0.17	-2.47	-1.78
2 months - 12 months	-2.00	0.24	-2.48	-1.53
3 months - 6 months	-1.75	0.15	-2.04	-1.46
3 months - 12 months	-1.62	0.22	-2.05	-1.20
6 months - 12 months	0.12	0.25	-0.37	0.61

SE: standard error of the Effect size estimate. CL: Confidence level. df = 895.

S7 Table. Estimated marginal means (EMM), pairwise mean differences, and effect sizes for half-width (a_2) across age groups.

a) Estimated marginal mean (EMM)

Age group	EMM	SE	Lower CL	Upper CL
2 months	6.83	0.04	6.74	6.91
3 months	6.96	0.02	6.91	7.00
6 months	6.87	0.06	6.76	6.99
12 months	7.67	0.10	7.48	7.86

SE: Standard error of the EMM. CL: Confidence limit. df = 895.

b) EMM difference

Contrast	EMM difference	SE	t ratio	P value	Significance
2 months - 3 months	-0.13	0.05	-2.50	0.02	*
2 months - 6 months	-0.04	0.08	-0.55	0.58	ns
2 months - 12 months	-0.84	0.11	-7.60	< 0.001	***
3 months - 6 months	0.09	0.07	1.32	0.22	ns
3 months - 12 months	-0.71	0.10	-7.17	< 0.001	***
6 months - 12 months	-0.79	0.12	-6.87	< 0.001	***

SE: standard error of the estimated difference between groups. df = 895.

c) Effect size

Contrast	Effect size	SE	Lower CL	Upper CL
2 months - 3 months	-0.28	0.11	-0.50	-0.06
2 months - 6 months	-0.09	0.17	-0.42	0.24
2 months - 12 months	-1.81	0.24	-2.29	-1.34
3 months - 6 months	0.19	0.14	-0.09	0.47
3 months - 12 months	-1.53	0.22	-1.96	-1.11
6 months - 12 months	-1.72	0.25	-2.22	-1.22

SE: standard error of the Effect size estimate. CL: Confidence level. df = 895.

S8 Table. Estimated marginal means (EMM), pairwise mean differences, and effect sizes for height(*h*) across age groups.

a) Estimated marginal mean (EMM)

	EMM	SE	Lower CL	Upper CL
2 months	8.02	0.05	7.92	8.11
3 months	8.44	0.02	8.39	8.49
6 months	9.17	0.06	9.05	9.30
12 months	9.91	0.11	9.71	10.12

SE: Standard error of the EMM. CL: Confidence limit. df = 895.

b) EMM difference

Contrast	EMM difference	SE	t ratio	P value	Significance
2 months - 3 months	-0.42	0.06	-7.49	< 0.001	***
2 months - 6 months	-1.16	0.08	-13.70	< 0.001	***
2 months - 12 months	-1.90	0.12	-15.75	< 0.001	***
3 months - 6 months	-0.73	0.07	-10.22	< 0.001	***
3 months - 12 months	-1.47	0.11	-13.64	< 0.001	***
6 months - 12 months	-0.74	0.13	-5.84	< 0.001	***

SE: standard error of the estimated difference between groups. df = 895.

c) Effect size

Contrast	Effect size	SE	Lower CL	Upper CL
2 months - 3 months	-0.84	0.11	-1.06	-0.62
2 months - 6 months	-2.29	0.18	-2.63	-1.94
2 months - 12 months	-3.75	0.25	-4.25	-3.25
3 months - 6 months	-1.45	0.15	-1.74	-1.16
3 months - 12 months	-2.91	0.22	-3.35	-2.47
6 months - 12 months	-1.46	0.25	-1.96	-0.97

SE: standard error of the Effect size estimate. CL: Confidence level. df = 895.

S9 Table. Estimated marginal means (EMM), pairwise mean differences, and effect sizes for cephalic index (CI) across age groups.

a) Estimated marginal mean (EMM)

Age group	EMM	SE	Lower CL	Upper CL
2 months	87.87	1.12	85.68	90.07
3 months	88.53	0.58	87.39	89.67
6 months	100.57	1.49	97.64	103.50
12 months	90.12	2.50	85.22	95.02

SE: Standard error of the EMM. CL: Confidence limit. df = 895.

b) EMM difference

Contrast	EMM difference	SE	t ratio	P value	Significance
2 months - 3 months	-0.66	1.33	-0.49	0.62	ns
2 months - 6 months	-12.69	1.98	-6.41	< 0.001	***
2 months - 12 months	-2.25	2.82	-0.80	0.62	ns
3 months - 6 months	-12.04	1.68	-7.16	< 0.001	***
3 months - 12 months	-1.59	2.53	-0.63	0.62	ns
6 months - 12 months	10.45	2.97	3.52	0.001	***

SE: standard error of the estimated difference between groups. df = 895.

c) Effect size

Contrast	Effect size	SE	Lower CL	Upper CL
2 months - 3 months	-0.06	0.11	-0.28	0.16
2 months - 6 months	-1.07	0.17	-1.40	-0.74
2 months - 12 months	-0.19	0.24	-0.66	0.28
3 months - 6 months	-1.02	0.14	-1.30	-0.73
3 months - 12 months	-0.13	0.21	-0.55	0.29
6 months - 12 months	0.88	0.25	0.39	1.37

SE: standard error of the Effect size estimate. CL: Confidence level. df = 895.

S10 Table. Estimated marginal means (EMM), pairwise mean differences, and effect sizes for height-half length ratio across age groups.

a) Estimated marginal mean (EMM)

Age group	EMM	SE	Lower CL	Upper CL
2 months	1.18	0.01	1.16	1.20
3 months	1.22	0.004	1.21	1.23
6 months	1.34	0.01	1.31	1.36
12 months	1.30	0.02	1.26	1.34

SE: Standard error of the EMM. CL: Confidence limit. df = 895.

c) EMM difference

Contrast	EMM difference	SE	t ratio	P value	Significance
2 months - 3 months	-0.04	0.01	-3.67	< 0.001	***
2 months - 6 months	-0.16	0.02	-9.71	< 0.001	***
2 months - 12 months	-0.12	0.02	-5.20	< 0.001	***
3 months - 6 months	-0.12	0.01	-8.54	< 0.001	***
3 months - 12 months	-0.08	0.02	-3.87	< 0.001	***
6 months - 12 months	0.04	0.02	1.53	0.13	ns

SE: standard error of the estimated difference between groups. df = 895.

d) Effect size

Contrast	Effect size	SE	Lower CL	Upper CL
2 months - 3 months	-0.41	0.11	-0.63	-0.19
2 months - 6 months	-1.62	0.17	-1.96	-1.29
2 months - 12 months	-1.24	0.24	-1.71	-0.77
3 months - 6 months	-1.21	0.14	-1.49	-0.93
3 months - 12 months	-0.83	0.21	-1.25	-0.41
6 months - 12 months	0.38	0.25	-0.11	0.87

SE: standard error of the Effect size estimate. CL: Confidence level. df = 895.

S11 Table. Estimated marginal means (EMM), pairwise mean differences, and effect sizes for volume across age groups.

a) Estimated marginal mean (EMM)

Age group	EMM	SE	Lower CL	Upper CL
2 months	677.14	6.06	665.24	689.04
3 months	747.41	3.14	741.24	753.59
6 months	909.93	8.08	894.07	925.78
12 months	1086.17	13.53	1059.61	1112.72

SE: Standard error of the EMM. CL: Confidence limit. df = 895.

b) EMM difference

Contrast	EMM difference	SE	t ratio	P value	Significance
2 months - 3 months	-70.28	7.20	-9.77	< 0.001	***
2 months - 6 months	-232.79	10.72	-21.71	< 0.001	***
2 months - 12 months	-409.03	15.30	-26.74	< 0.001	***
3 months - 6 months	-162.51	9.10	-17.85	< 0.001	***
3 months - 12 months	-338.75	13.71	-24.70	< 0.001	***
6 months - 12 months	-176.24	16.08	-10.96	< 0.001	***

SE: standard error of the estimated difference between groups. df = 895.

c) Effect size

Contrast	Effect size	SE	Lower CL	Upper CL
2 months - 3 months	-1.09	0.12	-1.32	-0.87
2 months - 6 months	-3.63	0.19	-3.99	-3.26
2 months - 12 months	-6.37	0.28	-6.92	-5.82
3 months - 6 months	-2.53	0.15	-2.83	-2.23
3 months - 12 months	-5.28	0.25	-5.76	-4.79
6 months - 12 months	-2.75	0.26	-3.25	-2.24

SE: standard error of the Effect size estimate. CL: Confidence level. df = 895.

S12 Table. Estimated marginal means (EMM), pairwise mean differences, and effect sizes for globularity index (GI) across age groups.

a) Estimated marginal mean (EMM)

Age group	EMM	SE	Lower CL	Upper CL
2 months	0.984	0.001	0.982	0.986
3 months	0.982	4.58×10^{-4}	0.981	0.983
6 months	0.986	0.001	0.984	0.988
12 months	0.979	0.002	0.975	0.982

SE: Standard error of the EMM. CL: Confidence limit. df = 895.

Values are displayed to three decimal places as rounding up to two decimal places either results in zero or the same value across most entries.

Values < 0.001 are reported in scientific notation with three significant figures.

b) EMM difference

Contrast	EMM difference	SE	t ratio	P value	Significance
2 months - 3 months	0.002	0.001	1.68	0.11	ns
2 months - 6 months	-0.002	0.002	-1.25	0.21	ns
2 months - 12 months	0.005	0.002	2.44	0.03	*
3 months - 6 months	-0.004	0.001	-2.80	0.02	*
3 months - 12 months	0.004	0.002	1.84	0.10	ns
6 months - 12 months	0.007	0.002	3.15	0.01	*

SE: standard error of the estimated difference between groups. df = 895.

Values are displayed to three decimal places for EMM difference and SE as rounding up to two decimal places result in zero for most entries.

c) Effect size

Contrast	Effect size	SE	Lower CL	Upper CL
2 months - 3 months	0.19	0.11	-0.03	0.41
2 months - 6 months	-0.21	0.17	-0.54	0.12
2 months - 12 months	0.58	0.24	0.11	1.05
3 months - 6 months	-0.40	0.14	-0.68	-0.12
3 months - 12 months	0.39	0.21	-0.03	0.81
6 months - 12 months	0.79	0.25	0.30	1.28

SE: standard error of the Effect size estimate. CL: Confidence level. df = 895.

S13 Table. Estimated marginal means (EMM) by sex within each month group and sex differences (mean difference and effect size) for Left Tragion-Right Tragion (LT-RT) across age groups and sex.

a) Estimated marginal mean (EMM)

	Sex	EMM	SE	Lower CL	Upper CL
2 months	Female	26.96	0.12	26.72	27.20
	Male	27.81	0.13	27.56	28.06
3 months	Female	28.13	0.07	27.98	28.27
	Male	28.98	0.07	28.83	29.12
6 months	Female	31.10	0.17	30.78	31.43
	Male	31.96	0.16	31.64	32.27
12 months	Female	32.45	0.27	31.92	32.98
	Male	33.30	0.26	32.78	33.82

SE: Standard error of the EMM. CL: Confidence limit. df = 895

b) EMM difference

Contrast	estimate	SE	t ratio	P value	Significance
Female – Male	-0.85	0.08	-10.09	< 0.001	***

SE: standard error of the estimated difference between groups. df = 895.

EMM differences are identical across age groups.

c) Effect size

Contrast	Effect size	SE	Lower CL	Upper CL
Female – Male	-0.68	0.07	-0.82	-0.54

SE: standard error of the effect size estimate. CL: Confidence level. df = 895.

Effect sizes are identical across age groups.

S14 Table. Estimated marginal means (EMM) by sex within each month group and sex differences (mean difference and effect size) for Glabella-Occipital protuberance (G-O).

a) Estimated marginal mean (EMM)

	Sex	EMM	SE	Lower CL	Upper CL
2 months	Female	23.08	0.12	22.85	23.32
	Male	23.55	0.12	23.32	23.79
3 months	Female	23.95	0.07	23.81	24.09
	Male	24.42	0.07	24.29	24.56
6 months	Female	24.97	0.16	24.66	25.28
	Male	25.44	0.15	25.14	25.74
12 months	Female	27.38	0.26	26.88	27.88
	Male	27.85	0.25	27.36	28.35

SE: Standard error of the EMM. CL: Confidence limit. df = 895

b) EMM difference

Contrast	EMM difference	SE	t ratio	P value	Significance
Female – Male	-0.47	0.08	-5.85	< 0.001	***

SE: standard error of the estimated difference between groups. df = 895.

EMM differences are identical across age groups.

c) Effect size

Contrast	Effect size	SE	Lower CL	Upper CL
Female – Male	-0.39	0.07	-0.53	-0.26

SE: standard error of the effect size estimate. CL: Confidence level. df = 895.

Effect sizes are identical across age groups.

S15 Table. Estimated marginal means (EMM) by sex within each month group and sex differences (mean difference and effect size) for head circumference (HC).

a) Estimated marginal mean (EMM)

Age group	Sex	EMM	SE	Lower CL	Upper CL
2 months	Female	39.64	0.10	39.44	39.84
	Male	40.53	0.10	40.33	40.74
3 months	Female	40.58	0.06	40.46	40.70
	Male	41.47	0.06	41.35	41.59
6 months	Female	42.77	0.14	42.50	43.04
	Male	43.66	0.13	43.40	43.92
12 months	Female	45.09	0.22	44.66	45.53
	Male	45.98	0.22	45.56	46.41

SE: Standard error of the EMM. CL: Confidence limit. df = 895

b) EMM difference

Contrast	EMM difference	SE	t ratio	P value	Significance
Female – Male	-0.89	0.07	-12.91	< 0.001	***

SE: standard error of the estimated difference between groups. df = 895.

EMM differences are identical across age groups.

c) Effect size

Contrast	Effect size	SE	Lower CL	Upper CL
Female – Male	-0.87	0.07	-1.01	-0.73

SE: standard error of the Effect size estimate. CL: Confidence level. df = 895.

Effect sizes are identical across age groups.

S16 Table. Estimated marginal means (EMM) by sex within each month group and sex differences (mean difference and effect size) for half-width (a_1).

a) Estimated marginal mean (EMM)

	Sex	EMM	SE	Lower CL	Upper CL
2 months	Female	5.83	0.04	5.74	5.91
	Male	6.04	0.05	5.95	6.12
3 months	Female	6.00	0.03	5.94	6.05
	Male	6.21	0.03	6.15	6.26
6 months	Female	6.78	0.06	6.66	6.90
	Male	6.99	0.06	6.88	7.10
12 months	Female	6.73	0.10	6.54	6.91
	Male	6.93	0.10	6.75	7.12

SE: Standard error of the EMM. CL: Confidence limit. df = 895

b) EMM difference

Contrast	EMM difference	SE	t ratio	P value	Significance
Female – Male	-0.21	0.03	-6.91	< 0.001	***

SE: standard error of the estimated difference between groups. df = 895.

EMM differences are identical across age groups.

c) Effect size

Contrast	Effect size	SE	Lower CL	Upper CL
Female – Male	-0.47	0.07	-0.60	-0.33

SE: standard error of the effect size estimate. CL: Confidence level. df = 895.

S17 Table. Estimated marginal means (EMM) by sex within each month group and sex differences (mean difference and effect size) for half-length (a_2).

a) Estimated marginal mean (EMM)

	Sex	EMM	SE	Lower CL	Upper CL
2 months	Female	6.79	0.05	6.70	6.88
	Male	6.87	0.05	6.77	6.96
3 months	Female	6.92	0.03	6.87	6.98
	Male	7.00	0.03	6.94	7.05
6 months	Female	6.83	0.06	6.71	6.95
	Male	6.91	0.06	6.79	7.02
12 months	Female	7.63	0.10	7.43	7.82
	Male	7.70	0.10	7.51	7.90

SE: Standard error of the EMM. CL: Confidence limit. df = 895

b) EMM difference

Contrast	EMM difference	SE	t ratio	P value	Significance
Female – Male	-0.07	0.03	-2.41	0.02	*

SE: standard error of the estimated difference between groups. df = 895.

EMM differences are identical across age groups.

c) Effect size

Contrast	Effect size	SE	Lower CL	Upper CL
Female - Male	-0.16	0.07	-0.29	-0.03

SE: standard error of the effect size estimate. CL: Confidence level. df = 895.

Effect sizes are identical across age groups.

S18 Table. Estimated marginal means (EMM) by sex within each month group and sex differences (mean difference and effect size) for height(*h*).

a) Estimated marginal mean (EMM)

	Sex	EMM	SE	Lower CL	Upper CL
2 months	Female	7.90	0.05	7.81	8.00
	Male	8.13	0.05	8.03	8.23
3 months	Female	8.33	0.03	8.27	8.39
	Male	8.55	0.03	8.49	8.61
6 months	Female	9.06	0.07	8.93	9.19
	Male	9.29	0.06	9.16	9.41
12 months	Female	9.80	0.11	9.59	10.02
	Male	10.03	0.11	9.82	10.24

SE: Standard error of the EMM. CL: Confidence limit. df = 895

b) EMM difference

Contrast	EMM difference	SE	t ratio	P value	Significance
Female – Male	-0.22	0.03	-6.58	< 0.001	***

SE: standard error of the estimated difference between groups. df = 895.

EMM differences are identical across age groups.

c) Effect size

Contrast	Effect size	SE	Lower CL	Upper CL
Female - Male	-0.44	0.07	-0.58	-0.31

SE: standard error of the Effect size estimate. CL: Confidence level. df = 895.

Effect sizes are identical across age groups.

S19 Table. Estimated marginal means (EMM) by sex within each month group and sex differences (mean difference and effect size) for cephalic index (CI).

a) Estimated marginal mean (EMM)

	Sex	EMM	SE	Lower CL	Upper CL
2 months	Female	86.78	1.17	84.47	89.08
	Male	88.97	1.20	86.61	91.33
3 months	Female	87.43	0.71	86.04	88.82
	Male	89.63	0.70	88.25	91.00
6 months	Female	99.47	1.58	96.38	102.56
	Male	101.67	1.51	98.70	104.63
12 months	Female	89.02	2.55	84.02	94.03
	Male	91.22	2.51	86.29	96.14

SE: Standard error of the EMM. CL: Confidence limit. df = 895

b) EMM difference

Contrast	EMM difference	SE	t ratio	P value	Significance
Female – Male	-2.19	0.80	-2.75	0.01	**

SE: standard error of the estimated difference between groups. df = 895.

EMM differences are identical across age groups.

c) Effect size

Contrast	Effect size	SE	Lower CL	Upper CL
Female – Male	-0.19	0.07	-0.32	-0.05

SE: standard error of the effect size estimate. CL: Confidence level. df = 895.

Effect sizes are identical across age groups.

S20 Table. Estimated marginal means (EMM) by sex within each month group and sex differences (mean difference and effect size) for height-half length ratio (h/a_2).

a) Estimated marginal mean (EMM)

	Sex	EMM	SE	Lower CL	Upper CL
2 months	Female	1.17	0.01	1.15	1.19
	Male	1.19	0.01	1.17	1.21
3 months	Female	1.21	0.01	1.20	1.22
	Male	1.23	0.01	1.22	1.24
6 months	Female	1.33	0.01	1.30	1.35
	Male	1.35	0.01	1.32	1.37
12 months	Female	1.29	0.02	1.25	1.33
	Male	1.31	0.02	1.27	1.35

SE: Standard error of the EMM. CL: Confidence limit. df = 895

b) EMM difference

Contrast	EMM difference	SE	t ratio	P value	Significance
Female – Male	-0.02	0.01	-2.99	0.003	**

SE: standard error of the estimated difference between groups. df = 895.

EMM differences are identical across age groups.

c) Effect size

Contrast	Effect size	SE	Lower CL	Upper CL
Female – Male	-0.20	0.07	-0.33	-0.07

SE: standard error of the effect size estimate. CL: Confidence level. df = 895.

Effect sizes are identical across age groups.

S21 Table. Estimated marginal means (EMM) by sex within each month group and sex differences (mean difference and effect size) for volume.

a) Estimated marginal mean (EMM)

	Sex	EMM	SE	Lower CL	Upper CL
2 months	Female	650.35	6.36	637.87	662.83
	Male	703.93	6.51	691.14	716.71
3 months	Female	720.63	3.83	713.10	728.15
	Male	774.20	3.80	766.75	781.66
6 months	Female	883.14	8.54	866.38	899.89
	Male	936.72	8.19	920.65	952.78
12 months	Female	1059.38	13.81	1032.27	1086.48
	Male	1112.95	13.59	1086.27	1139.64

SE: Standard error of the EMM. CL: Confidence limit. df = 895

b) EMM difference

Contrast	EMM difference	SE	t ratio	P value	Significance
Female – Male	-53.58	4.32	-12.39	< 0.001	***

SE: standard error of the estimated difference between groups. df = 895.

EMM differences are identical across age groups.

c) Effect size

Contrast	Effect size	SE	Lower CL	Upper CL
Female - Male	-0.83	0.07	-0.97	-0.70

SE: standard error of the effect size estimate. CL: Confidence level. df = 895.

Effect sizes are identical across age groups.

S22 Table. Estimated marginal means (EMM) by sex within each month group and sex differences (mean difference and effect size) for globularity index (GI).

a) Estimated marginal mean (EMM)

	Sex	EMM	SE	Lower CL	Upper CL
2 months	Female	0.984	0.001	0.982	0.986
	Male	0.984	0.001	0.982	0.986
3 months	Female	0.982	0.001	0.981	0.983
	Male	0.982	0.001	0.981	0.983
6 months	Female	0.986	0.001	0.984	0.988
	Male	0.986	0.001	0.983	0.988
12 months	Female	0.979	0.002	0.975	0.983
	Male	0.978	0.002	0.974	0.982

SE: Standard error of the EMM. CL: Confidence limit. df = 895

Values are displayed to three decimal places as rounding up to two decimal places either results in zero or the same value across most entries.

b) EMM difference

Contrast	EMM difference	SE	t ratio	P value	Significance
Female – Male	3.0×10^{-4}	6.3×10^{-4}	0.48	0.63	ns

SE: standard error of the estimated difference between groups. df = 895.

EMM differences are identical across age groups.

EMM difference and SE are reported in scientific notation with two significant figures as rounding up to two decimal places results in zero.

Values < 0.001 are reported in scientific notation with three significant figures.

c) Effect size

Contrast	Effect size	SE	Lower CL	Upper CL
Female - Male	0.03	0.07	-0.10	0.16

SE: standard error of the effect size estimate. CL: Confidence level. df = 895.

Effect sizes are identical across age groups.

S23 Table. ANOVA tables for estimated measurements collected at 3 months.

a) Half-width (a_1)

	Sum Sq	F value	P value	Significance
Birth year	16.53	9.44	< 0.001	***
Sex	3.73	19.19	< 0.001	***
Residuals	109.34			

b) Half-length (a_2)

	Sum Sq	F value	P value	Significance
Birth year	24.52	13.75	< 0.001	***
Sex	1.35	6.79	0.009	**
Residuals	111.37			

c) Height (h)

	Sum Sq	F value	P value	Significance
Birth year	9.27	3.97	< 0.001	***
Sex	6.93	26.71	< 0.001	***
Residuals	145.80			

d) Cephalic index (CI)

	Sum Sq	F value	P value	Significance
Birth year	14503.06	12.60	< 0.001	***
Sex	186.08	1.46	0.23	ns
Residuals	71860.61			

e) Globularity index (GI)

	Sum Sq	F value	P value	Significance
Birth year	0.002	2.78	0.003	***
Sex	1.5×10^{-5}	0.17	0.68	ns
Residuals	0.051			

f) Height-half-length ratio (h/a_2)

	Sum Sq	F value	P value	Significance
Birth year	1.34	16.70	< 0.001	***
Sex	0.03	3.41	0.07	ns
Residuals	5.00			

g) Volume

	Sum Sq	F value	P value	Significance
Birth year	52970.25	1.41	0.18	ns
Sex	353591.59	84.93	< 0.001	***
Residuals	2339724.66			

Sum Sq: Sum of squares.

For all tables, df = 3 for age (months), df = 9 for birth year, df = 1 for sex, df = 895 for residuals.

For GI, sum of squares are displayed to three decimal places if rounding up to two decimal places results in zero, and values < 0.001 are reported in scientific notation with two significant figures.

S24 Table. Estimated marginal mean (EMM) of half-width (a_1) across birth year groups

Birth year	EMM	SE	Lower CL	Upper CL
2010	6.22	0.06	6.10	6.34
2011	6.28	0.04	6.19	6.37
2012	6.18	0.04	6.11	6.25
2013	6.28	0.09	6.11	6.45
2014	6.33	0.06	6.21	6.45
2015	6.28	0.11	6.05	6.50
2016	5.69	0.10	5.49	5.89
2017	5.94	0.06	5.82	6.06
2018	5.89	0.06	5.78	5.99
2019	5.95	0.06	5.83	6.08

SE: Standard error of the EMM. CL: Confidence limit. df = 562

S25 Table. Pairwise comparisons of estimated marginal means (EMM) between birth year groups for half-width (a_1).

Contrast	EMM difference	SE	t ratio	P value	Significance
birth_year2010 - birth_year2011	-0.06	0.08	-0.75	0.60	ns
birth_year2010 - birth_year2012	0.04	0.07	0.61	0.66	ns
birth_year2010 - birth_year2013	-0.06	0.11	-0.54	0.70	ns
birth_year2010 - birth_year2014	-0.11	0.09	-1.26	0.31	ns
birth_year2010 - birth_year2015	-0.05	0.13	-0.43	0.74	ns
birth_year2010 - birth_year2016	0.53	0.12	4.48	< 0.001	***
birth_year2010 - birth_year2017	0.28	0.09	3.24	0.003	**
birth_year2010 - birth_year2018	0.34	0.08	4.07	< 0.001	***
birth_year2010 - birth_year2019	0.27	0.09	3.02	0.006	**
birth_year2011 - birth_year2012	0.10	0.06	1.76	0.13	ns
birth_year2011 - birth_year2013	0.001	0.10	-0.01	0.99	ns
birth_year2011 - birth_year2014	-0.05	0.08	-0.69	0.63	ns
birth_year2011 - birth_year2015	0.002	0.12	0.02	0.99	ns
birth_year2011 - birth_year2016	0.59	0.11	5.32	< 0.001	***
birth_year2011 - birth_year2017	0.34	0.08	4.46	< 0.001	***
birth_year2011 - birth_year2018	0.39	0.07	5.54	< 0.001	***
birth_year2011 - birth_year2019	0.32	0.08	4.17	< 0.001	***
birth_year2012 - birth_year2013	-0.10	0.10	-1.06	0.42	ns
birth_year2012 - birth_year2014	-0.15	0.07	-2.14	0.06	ns
birth_year2012 - birth_year2015	-0.10	0.12	-0.82	0.57	ns
birth_year2012 - birth_year2016	0.49	0.11	4.53	< 0.001	***
birth_year2012 - birth_year2017	0.24	0.07	3.33	0.003	**
birth_year2012 - birth_year2018	0.29	0.07	4.41	< 0.001	***
birth_year2012 - birth_year2019	0.22	0.07	3.04	0.006	**
birth_year2013 - birth_year2014	-0.05	0.11	-0.48	0.73	ns
birth_year2013 - birth_year2015	0.003	0.14	0.02	0.99	ns
birth_year2013 - birth_year2016	0.59	0.13	4.38	< 0.001	***
birth_year2013 - birth_year2017	0.34	0.11	3.16	0.004	**
birth_year2013 - birth_year2018	0.39	0.10	3.78	< 0.001	***
birth_year2013 - birth_year2019	0.33	0.11	2.99	0.006	**
birth_year2014 - birth_year2015	0.05	0.13	0.42	0.74	ns
birth_year2014 - birth_year2016	0.64	0.12	5.39	< 0.001	***
birth_year2014 - birth_year2017	0.39	0.09	4.47	< 0.001	***
birth_year2014 - birth_year2018	0.45	0.08	5.36	< 0.001	***
birth_year2014 - birth_year2019	0.38	0.09	4.21	< 0.001	***
birth_year2015 - birth_year2016	0.59	0.15	3.84	< 0.001	***
birth_year2015 - birth_year2017	0.34	0.13	2.60	0.02	*
birth_year2015 - birth_year2018	0.39	0.13	3.09	0.005	**
birth_year2015 - birth_year2019	0.32	0.13	2.47	0.03	*
birth_year2016 - birth_year2017	-0.25	0.12	-2.09	0.06	ns
birth_year2016 - birth_year2018	-0.19	0.12	-1.68	0.14	ns
birth_year2016 - birth_year2019	-0.26	0.12	-2.20	0.05	ns
birth_year2017 - birth_year2018	0.05	0.08	0.65	0.65	ns
birth_year2017 - birth_year2019	-0.01	0.09	-0.16	0.93	ns
birth_year2018 - birth_year2019	-0.07	0.08	-0.81	0.57	ns

SE: standard error of the estimated difference between groups. df = 562.

EMM differences are displayed to three decimal places if rounding up to two decimal places results in zero.

S26 Table. Effect sizes for pairwise estimated marginal mean (EMM) comparisons between birth year groups for half-width (a_1).

Contrast	Effect size	SE	Lower CL	Upper CL
birth_year2010 - birth_year2011	-0.13	0.17	-0.46	0.21
birth_year2010 - birth_year2012	0.10	0.16	-0.22	0.42
birth_year2010 - birth_year2013	-0.13	0.24	-0.61	0.35
birth_year2010 - birth_year2014	-0.25	0.20	-0.63	0.14
birth_year2010 - birth_year2015	-0.12	0.29	-0.70	0.45
birth_year2010 - birth_year2016	1.20	0.27	0.67	1.73
birth_year2010 - birth_year2017	0.64	0.20	0.25	1.03
birth_year2010 - birth_year2018	0.76	0.19	0.39	1.13
birth_year2010 - birth_year2019	0.61	0.20	0.21	1.00
birth_year2011 - birth_year2012	0.23	0.13	-0.03	0.48
birth_year2011 - birth_year2013	-0.002	0.22	-0.44	0.44
birth_year2011 - birth_year2014	-0.12	0.17	-0.46	0.22
birth_year2011 - birth_year2015	0.004	0.28	-0.54	0.55
birth_year2011 - birth_year2016	1.33	0.25	0.83	1.83
birth_year2011 - birth_year2017	0.77	0.17	0.43	1.11
birth_year2011 - birth_year2018	0.89	0.16	0.57	1.21
birth_year2011 - birth_year2019	0.74	0.18	0.39	1.08
birth_year2012 - birth_year2013	-0.23	0.22	-0.65	0.20
birth_year2012 - birth_year2014	-0.35	0.16	-0.67	-0.03
birth_year2012 - birth_year2015	-0.22	0.27	-0.76	0.31
birth_year2012 - birth_year2016	1.10	0.25	0.62	1.59
birth_year2012 - birth_year2017	0.54	0.16	0.22	0.86
birth_year2012 - birth_year2018	0.66	0.15	0.36	0.96
birth_year2012 - birth_year2019	0.51	0.17	0.18	0.84
birth_year2013 - birth_year2014	-0.12	0.24	-0.60	0.36
birth_year2013 - birth_year2015	0.01	0.33	-0.64	0.65
birth_year2013 - birth_year2016	1.33	0.31	0.73	1.94
birth_year2013 - birth_year2017	0.77	0.25	0.29	1.25
birth_year2013 - birth_year2018	0.89	0.24	0.43	1.36
birth_year2013 - birth_year2019	0.74	0.25	0.25	1.22
birth_year2014 - birth_year2015	0.12	0.29	-0.45	0.70
birth_year2014 - birth_year2016	1.45	0.27	0.92	1.99
birth_year2014 - birth_year2017	0.89	0.20	0.49	1.28
birth_year2014 - birth_year2018	1.01	0.19	0.63	1.38
birth_year2014 - birth_year2019	0.85	0.20	0.45	1.26
birth_year2015 - birth_year2016	1.33	0.35	0.64	2.01
birth_year2015 - birth_year2017	0.76	0.29	0.19	1.34
birth_year2015 - birth_year2018	0.89	0.29	0.32	1.45
birth_year2015 - birth_year2019	0.73	0.30	0.15	1.31
birth_year2016 - birth_year2017	-0.56	0.27	-1.09	-0.03
birth_year2016 - birth_year2018	-0.44	0.26	-0.96	0.07
birth_year2016 - birth_year2019	-0.60	0.27	-1.13	-0.06
birth_year2017 - birth_year2018	0.12	0.19	-0.25	0.49
birth_year2017 - birth_year2019	-0.03	0.20	-0.43	0.36
birth_year2018 - birth_year2019	-0.16	0.19	-0.53	0.22

SE: standard error of the effect size estimate. CL: Confidence level. df = 562.

Effect sizes are displayed to three decimal places if rounding up to two decimal places results in zero.

S27 Table. Estimated marginal mean (EMM) of half-length (a_2) for each birth year.

Birth year	EMM	SE	Lower CL	Upper CL
2010	6.97	0.06	6.85	7.09
2011	6.73	0.04	6.64	6.82
2012	6.86	0.04	6.79	6.93
2013	6.72	0.09	6.54	6.89
2014	6.64	0.06	6.52	6.77
2015	6.74	0.11	6.51	6.96
2016	7.42	0.10	7.22	7.62
2017	7.15	0.06	7.03	7.27
2018	7.22	0.06	7.11	7.33
2019	7.10	0.06	6.98	7.23

SE: Standard error of the EMM. CL: Confidence limit. df = 562

S28 Table. Pairwise comparisons of estimated marginal means (EMM) between birth year groups for half-length (a_2).

Contrast	EMM difference	SE	t ratio	P value	Significance
birth_year2010 - birth_year2011	0.24	0.08	3.17	0.003	**
birth_year2010 - birth_year2012	0.11	0.07	1.59	0.15	ns
birth_year2010 - birth_year2013	0.26	0.11	2.36	0.03	*
birth_year2010 - birth_year2014	0.33	0.09	3.74	< 0.001	***
birth_year2010 - birth_year2015	0.23	0.13	1.79	0.11	ns
birth_year2010 - birth_year2016	-0.45	0.12	-3.78	< 0.001	***
birth_year2010 - birth_year2017	-0.18	0.09	-2.06	0.06	ns
birth_year2010 - birth_year2018	-0.25	0.08	-3.01	0.005	**
birth_year2010 - birth_year2019	-0.13	0.09	-1.50	0.18	ns
birth_year2011 - birth_year2012	-0.13	0.06	-2.21	0.04	*
birth_year2011 - birth_year2013	0.01	0.10	0.14	0.91	ns
birth_year2011 - birth_year2014	0.09	0.08	1.13	0.31	ns
birth_year2011 - birth_year2015	-0.01	0.12	-0.07	0.95	ns
birth_year2011 - birth_year2016	-0.69	0.11	-6.21	< 0.001	***
birth_year2011 - birth_year2017	-0.42	0.08	-5.51	< 0.001	***
birth_year2011 - birth_year2018	-0.49	0.07	-6.88	< 0.001	***
birth_year2011 - birth_year2019	-0.38	0.08	-4.78	< 0.001	***
birth_year2012 - birth_year2013	0.14	0.10	1.47	0.18	ns
birth_year2012 - birth_year2014	0.21	0.07	2.97	0.006	**
birth_year2012 - birth_year2015	0.12	0.12	0.99	0.38	ns
birth_year2012 - birth_year2016	-0.56	0.11	-5.20	< 0.001	***
birth_year2012 - birth_year2017	-0.29	0.07	-4.08	< 0.001	***
birth_year2012 - birth_year2018	-0.36	0.07	-5.44	< 0.001	***
birth_year2012 - birth_year2019	-0.25	0.07	-3.33	0.002	**
birth_year2013 - birth_year2014	0.07	0.11	0.67	0.55	ns
birth_year2013 - birth_year2015	-0.02	0.15	-0.15	0.91	ns
birth_year2013 - birth_year2016	-0.71	0.14	-5.21	< 0.001	***
birth_year2013 - birth_year2017	-0.44	0.11	-4.02	< 0.001	***
birth_year2013 - birth_year2018	-0.51	0.11	-4.81	< 0.001	***
birth_year2013 - birth_year2019	-0.39	0.11	-3.54	0.001	**
birth_year2014 - birth_year2015	-0.10	0.13	-0.73	0.53	ns
birth_year2014 - birth_year2016	-0.78	0.12	-6.51	< 0.001	***
birth_year2014 - birth_year2017	-0.51	0.09	-5.77	< 0.001	***
birth_year2014 - birth_year2018	-0.58	0.08	-6.90	< 0.001	***
birth_year2014 - birth_year2019	-0.46	0.09	-5.11	< 0.001	***
birth_year2015 - birth_year2016	-0.68	0.15	-4.45	< 0.001	***
birth_year2015 - birth_year2017	-0.41	0.13	-3.17	0.003	**
birth_year2015 - birth_year2018	-0.48	0.13	-3.78	< 0.001	***
birth_year2015 - birth_year2019	-0.37	0.13	-2.78	0.01	*
birth_year2016 - birth_year2017	0.27	0.12	2.25	0.04	*
birth_year2016 - birth_year2018	0.20	0.12	1.72	0.12	ns
birth_year2016 - birth_year2019	0.32	0.12	2.62	0.02	*
birth_year2017 - birth_year2018	-0.07	0.08	-0.83	0.47	ns
birth_year2017 - birth_year2019	0.05	0.09	0.53	0.64	ns
birth_year2018 - birth_year2019	0.12	0.09	1.37	0.21	ns

SE: standard error of the estimated difference between groups. df = 562.

S29 Table. Effect sizes for pairwise estimated marginal mean (EMM) comparisons between birth year groups for half-length (a_2).

Contrast	Effect size	SE	Lower CL	Upper CL
birth_year2010 - birth_year2011	0.54	0.17	0.21	0.88
birth_year2010 - birth_year2012	0.26	0.16	-0.06	0.57
birth_year2010 - birth_year2013	0.57	0.24	0.09	1.05
birth_year2010 - birth_year2014	0.74	0.20	0.35	1.13
birth_year2010 - birth_year2015	0.52	0.29	-0.05	1.10
birth_year2010 - birth_year2016	-1.01	0.27	-1.54	-0.48
birth_year2010 - birth_year2017	-0.41	0.20	-0.79	-0.02
birth_year2010 - birth_year2018	-0.56	0.19	-0.93	-0.19
birth_year2010 - birth_year2019	-0.30	0.20	-0.70	0.09
birth_year2011 - birth_year2012	-0.29	0.13	-0.54	-0.03
birth_year2011 - birth_year2013	0.03	0.22	-0.41	0.47
birth_year2011 - birth_year2014	0.19	0.17	-0.14	0.53
birth_year2011 - birth_year2015	-0.02	0.28	-0.56	0.53
birth_year2011 - birth_year2016	-1.55	0.25	-2.05	-1.05
birth_year2011 - birth_year2017	-0.95	0.17	-1.29	-0.61
birth_year2011 - birth_year2018	-1.11	0.16	-1.43	-0.78
birth_year2011 - birth_year2019	-0.84	0.18	-1.19	-0.49
birth_year2012 - birth_year2013	0.32	0.22	-0.11	0.74
birth_year2012 - birth_year2014	0.48	0.16	0.16	0.80
birth_year2012 - birth_year2015	0.27	0.27	-0.26	0.80
birth_year2012 - birth_year2016	-1.27	0.25	-1.75	-0.78
birth_year2012 - birth_year2017	-0.66	0.16	-0.98	-0.34
birth_year2012 - birth_year2018	-0.82	0.15	-1.12	-0.52
birth_year2012 - birth_year2019	-0.56	0.17	-0.89	-0.23
birth_year2013 - birth_year2014	0.16	0.24	-0.32	0.64
birth_year2013 - birth_year2015	-0.05	0.33	-0.69	0.59
birth_year2013 - birth_year2016	-1.59	0.31	-2.19	-0.98
birth_year2013 - birth_year2017	-0.98	0.25	-1.46	-0.50
birth_year2013 - birth_year2018	-1.14	0.24	-1.61	-0.67
birth_year2013 - birth_year2019	-0.87	0.25	-1.36	-0.39
birth_year2014 - birth_year2015	-0.21	0.29	-0.79	0.36
birth_year2014 - birth_year2016	-1.75	0.27	-2.29	-1.21
birth_year2014 - birth_year2017	-1.14	0.20	-1.54	-0.75
birth_year2014 - birth_year2018	-1.30	0.19	-1.68	-0.92
birth_year2014 - birth_year2019	-1.04	0.21	-1.44	-0.63
birth_year2015 - birth_year2016	-1.54	0.35	-2.22	-0.85
birth_year2015 - birth_year2017	-0.93	0.30	-1.51	-0.35
birth_year2015 - birth_year2018	-1.09	0.29	-1.66	-0.52
birth_year2015 - birth_year2019	-0.82	0.30	-1.41	-0.24
birth_year2016 - birth_year2017	0.61	0.27	0.08	1.13
birth_year2016 - birth_year2018	0.45	0.26	-0.07	0.96
birth_year2016 - birth_year2019	0.71	0.27	0.18	1.25
birth_year2017 - birth_year2018	-0.16	0.19	-0.53	0.21
birth_year2017 - birth_year2019	0.11	0.20	-0.29	0.50
birth_year2018 - birth_year2019	0.26	0.19	-0.11	0.64

SE: standard error of the effect size estimate. CL: Confidence level. df = 562.

S30 Table. Estimated marginal mean (EMM) of height (*h*) for each birth year.

Birth year	EMM	SE	Lower CL	Upper CL
2010	8.24	0.07	8.10	8.38
2011	8.52	0.05	8.42	8.62
2012	8.58	0.04	8.50	8.66
2013	8.57	0.10	8.37	8.77
2014	8.53	0.07	8.39	8.67
2015	8.33	0.13	8.07	8.59
2016	8.07	0.12	7.84	8.30
2017	8.41	0.07	8.27	8.55
2018	8.41	0.06	8.28	8.53
2019	8.55	0.07	8.40	8.69

SE: Standard error of the EMM. CL: Confidence limit. df = 562

S31 Table. Pairwise comparisons of estimated marginal means (EMM) between birth year groups for height (*h*).

Contrast	EMM difference	SE	t ratio	P value	Significance
birth_year2010 - birth_year2011	-0.29	0.09	-3.28	0.008	**
birth_year2010 - birth_year2012	-0.34	0.08	-4.14	0.001	**
birth_year2010 - birth_year2013	-0.33	0.12	-2.66	0.04	*
birth_year2010 - birth_year2014	-0.29	0.10	-2.89	0.02	*
birth_year2010 - birth_year2015	-0.09	0.15	-0.62	0.72	ns
birth_year2010 - birth_year2016	0.17	0.14	1.23	0.33	ns
birth_year2010 - birth_year2017	-0.17	0.10	-1.69	0.24	ns
birth_year2010 - birth_year2018	-0.17	0.10	-1.76	0.22	ns
birth_year2010 - birth_year2019	-0.31	0.10	-3.01	0.02	*
birth_year2011 - birth_year2012	-0.05	0.07	-0.83	0.57	ns
birth_year2011 - birth_year2013	-0.04	0.11	-0.39	0.84	ns
birth_year2011 - birth_year2014	-0.004	0.09	-0.05	0.98	ns
birth_year2011 - birth_year2015	0.19	0.14	1.36	0.31	ns
birth_year2011 - birth_year2016	0.45	0.13	3.55	0.006	**
birth_year2011 - birth_year2017	0.12	0.09	1.32	0.31	ns
birth_year2011 - birth_year2018	0.12	0.08	1.43	0.31	ns
birth_year2011 - birth_year2019	-0.02	0.09	-0.26	0.90	ns
birth_year2012 - birth_year2013	0.01	0.11	0.10	0.97	ns
birth_year2012 - birth_year2014	0.05	0.08	0.61	0.72	ns
birth_year2012 - birth_year2015	0.25	0.14	1.79	0.22	ns
birth_year2012 - birth_year2016	0.51	0.12	4.09	0.001	**
birth_year2012 - birth_year2017	0.17	0.08	2.06	0.13	ns
birth_year2012 - birth_year2018	0.17	0.08	2.24	0.09	ns
birth_year2012 - birth_year2019	0.03	0.09	0.37	0.84	ns
birth_year2013 - birth_year2014	0.04	0.12	0.32	0.86	ns
birth_year2013 - birth_year2015	0.24	0.17	1.42	0.31	ns
birth_year2013 - birth_year2016	0.50	0.16	3.21	0.009	**
birth_year2013 - birth_year2017	0.16	0.12	1.29	0.32	ns
birth_year2013 - birth_year2018	0.16	0.12	1.34	0.31	ns
birth_year2013 - birth_year2019	0.02	0.13	0.17	0.93	ns
birth_year2014 - birth_year2015	0.20	0.15	1.32	0.31	ns
birth_year2014 - birth_year2016	0.46	0.14	3.34	0.008	**
birth_year2014 - birth_year2017	0.12	0.10	1.19	0.34	ns
birth_year2014 - birth_year2018	0.12	0.10	1.26	0.32	ns
birth_year2014 - birth_year2019	-0.02	0.10	-0.18	0.93	ns
birth_year2015 - birth_year2016	0.26	0.18	1.48	0.31	ns
birth_year2015 - birth_year2017	-0.08	0.15	-0.51	0.76	ns
birth_year2015 - birth_year2018	-0.08	0.15	-0.52	0.76	ns
birth_year2015 - birth_year2019	-0.22	0.15	-1.43	0.31	ns
birth_year2016 - birth_year2017	-0.34	0.14	-2.46	0.05	ns
birth_year2016 - birth_year2018	-0.34	0.13	-2.52	0.048	*
birth_year2016 - birth_year2019	-0.48	0.14	-3.45	0.007	**
birth_year2017 - birth_year2018	0.001	0.10	0.01	0.99	ns
birth_year2017 - birth_year2019	-0.14	0.10	-1.35	0.31	ns
birth_year2018 - birth_year2019	-0.14	0.10	-1.43	0.31	ns

SE: standard error of the estimated difference between groups. df = 562.

EMM differences are displayed to three decimal places if rounding up to two decimal places results in zero.

S32 Table. Effect sizes for pairwise estimated marginal mean (EMM) comparisons between birth year groups for height (*h*).

Contrast	Effect size	SE	Lower CL	Upper CL
birth_year2010 - birth_year2011	-0.56	0.17	-0.90	-0.22
birth_year2010 - birth_year2012	-0.67	0.16	-0.99	-0.35
birth_year2010 - birth_year2013	-0.65	0.24	-1.13	-0.17
birth_year2010 - birth_year2014	-0.57	0.20	-0.96	-0.18
birth_year2010 - birth_year2015	-0.18	0.29	-0.76	0.39
birth_year2010 - birth_year2016	0.33	0.27	-0.20	0.86
birth_year2010 - birth_year2017	-0.33	0.20	-0.72	0.06
birth_year2010 - birth_year2018	-0.33	0.19	-0.70	0.04
birth_year2010 - birth_year2019	-0.60	0.20	-1.00	-0.21
birth_year2011 - birth_year2012	-0.11	0.13	-0.36	0.15
birth_year2011 - birth_year2013	-0.09	0.22	-0.53	0.35
birth_year2011 - birth_year2014	-0.01	0.17	-0.35	0.33
birth_year2011 - birth_year2015	0.38	0.28	-0.17	0.92
birth_year2011 - birth_year2016	0.89	0.25	0.40	1.38
birth_year2011 - birth_year2017	0.23	0.17	-0.11	0.57
birth_year2011 - birth_year2018	0.23	0.16	-0.09	0.55
birth_year2011 - birth_year2019	-0.04	0.18	-0.39	0.30
birth_year2012 - birth_year2013	0.02	0.22	-0.40	0.45
birth_year2012 - birth_year2014	0.10	0.16	-0.22	0.42
birth_year2012 - birth_year2015	0.48	0.27	-0.05	1.02
birth_year2012 - birth_year2016	1.00	0.25	0.51	1.48
birth_year2012 - birth_year2017	0.33	0.16	0.02	0.65
birth_year2012 - birth_year2018	0.34	0.15	0.04	0.63
birth_year2012 - birth_year2019	0.06	0.17	-0.27	0.39
birth_year2013 - birth_year2014	0.08	0.24	-0.40	0.56
birth_year2013 - birth_year2015	0.46	0.33	-0.18	1.11
birth_year2013 - birth_year2016	0.98	0.31	0.38	1.58
birth_year2013 - birth_year2017	0.31	0.24	-0.17	0.79
birth_year2013 - birth_year2018	0.32	0.24	-0.15	0.78
birth_year2013 - birth_year2019	0.04	0.25	-0.44	0.53
birth_year2014 - birth_year2015	0.39	0.29	-0.19	0.96
birth_year2014 - birth_year2016	0.90	0.27	0.37	1.43
birth_year2014 - birth_year2017	0.24	0.20	-0.15	0.63
birth_year2014 - birth_year2018	0.24	0.19	-0.13	0.61
birth_year2014 - birth_year2019	-0.04	0.20	-0.44	0.36
birth_year2015 - birth_year2016	0.51	0.35	-0.17	1.19
birth_year2015 - birth_year2017	-0.15	0.29	-0.73	0.43
birth_year2015 - birth_year2018	-0.15	0.29	-0.71	0.42
birth_year2015 - birth_year2019	-0.42	0.30	-1.01	0.16
birth_year2016 - birth_year2017	-0.66	0.27	-1.19	-0.13
birth_year2016 - birth_year2018	-0.66	0.26	-1.18	-0.14
birth_year2016 - birth_year2019	-0.93	0.27	-1.47	-0.40
birth_year2017 - birth_year2018	0.001	0.19	-0.37	0.37
birth_year2017 - birth_year2019	-0.27	0.20	-0.67	0.12
birth_year2018 - birth_year2019	-0.27	0.19	-0.65	0.10

SE: standard error of the effect size estimate. CL: Confidence level. df = 562.

Effect sizes are displayed to three decimal places if rounding up to two decimal places results in zero.

S33 Table. Estimated marginal mean (EMM) of cephalic index (CI) for each birth year.

Birth year	EMM	SE	Lower CL	Upper CL
2010	90.24	1.57	87.16	93.32
2011	93.89	1.13	91.67	96.11
2012	90.77	0.93	88.94	92.60
2013	93.91	2.26	89.47	98.36
2014	95.96	1.58	92.84	99.07
2015	94.50	2.92	88.77	100.24
2016	77.31	2.59	72.21	82.40
2017	83.54	1.58	80.43	86.65
2018	81.93	1.42	79.14	84.73
2019	84.46	1.64	81.24	87.68

SE: Standard error of the EMM. CL: Confidence limit. df = 562

S34 Table. Pairwise comparisons of estimated marginal means (EMM) between birth year groups for cephalic index (CI).

Contrast	estimate	SE	t ratio	P value	Significance
birth_year2010 - birth_year2011	-3.65	1.93	-1.89	0.09	ns
birth_year2010 - birth_year2012	-0.53	1.82	-0.29	0.83	ns
birth_year2010 - birth_year2013	-3.67	2.75	-1.33	0.26	ns
birth_year2010 - birth_year2014	-5.71	2.23	-2.57	0.02	*
birth_year2010 - birth_year2015	-4.26	3.31	-1.29	0.26	ns
birth_year2010 - birth_year2016	12.94	3.03	4.27	< 0.001	***
birth_year2010 - birth_year2017	6.70	2.23	3.01	0.006	**
birth_year2010 - birth_year2018	8.31	2.12	3.92	< 0.001	***
birth_year2010 - birth_year2019	5.78	2.27	2.55	0.02	*
birth_year2011 - birth_year2012	3.12	1.46	2.13	0.05	ns
birth_year2011 - birth_year2013	-0.02	2.53	-0.01	0.99	ns
birth_year2011 - birth_year2014	-2.07	1.94	-1.06	0.35	ns
birth_year2011 - birth_year2015	-0.61	3.13	-0.20	0.88	ns
birth_year2011 - birth_year2016	16.58	2.83	5.86	< 0.001	***
birth_year2011 - birth_year2017	10.35	1.95	5.32	< 0.001	***
birth_year2011 - birth_year2018	11.96	1.82	6.57	< 0.001	***
birth_year2011 - birth_year2019	9.43	1.99	4.73	< 0.001	***
birth_year2012 - birth_year2013	-3.14	2.45	-1.28	0.26	ns
birth_year2012 - birth_year2014	-5.18	1.83	-2.83	0.009	**
birth_year2012 - birth_year2015	-3.73	3.06	-1.22	0.29	ns
birth_year2012 - birth_year2016	13.46	2.76	4.88	< 0.001	***
birth_year2012 - birth_year2017	7.23	1.84	3.93	< 0.001	***
birth_year2012 - birth_year2018	8.84	1.70	5.19	< 0.001	***
birth_year2012 - birth_year2019	6.31	1.89	3.34	0.002	**
birth_year2013 - birth_year2014	-2.04	2.76	-0.74	0.53	ns
birth_year2013 - birth_year2015	-0.59	3.69	-0.16	0.89	ns
birth_year2013 - birth_year2016	16.61	3.44	4.82	< 0.001	***
birth_year2013 - birth_year2017	10.37	2.76	3.76	< 0.001	***
birth_year2013 - birth_year2018	11.98	2.67	4.48	< 0.001	***
birth_year2013 - birth_year2019	9.45	2.79	3.38	0.002	**
birth_year2014 - birth_year2015	1.45	3.32	0.44	0.74	ns
birth_year2014 - birth_year2016	18.65	3.04	6.13	< 0.001	***
birth_year2014 - birth_year2017	12.41	2.24	5.54	< 0.001	***
birth_year2014 - birth_year2018	14.02	2.13	6.58	< 0.001	***
birth_year2014 - birth_year2019	11.49	2.30	5.01	< 0.001	***
birth_year2015 - birth_year2016	17.19	3.91	4.40	< 0.001	***
birth_year2015 - birth_year2017	10.96	3.32	3.30	0.002	**
birth_year2015 - birth_year2018	12.57	3.25	3.87	< 0.001	***
birth_year2015 - birth_year2019	10.04	3.35	3.00	0.006	**
birth_year2016 - birth_year2017	-6.24	3.04	-2.05	0.06	ns
birth_year2016 - birth_year2018	-4.63	2.96	-1.56	0.17	ns
birth_year2016 - birth_year2019	-7.15	3.07	-2.33	0.03	*
birth_year2017 - birth_year2018	1.61	2.13	0.76	0.53	ns
birth_year2017 - birth_year2019	-0.92	2.28	-0.40	0.75	ns
birth_year2018 - birth_year2019	-2.53	2.17	-1.16	0.31	ns

SE: standard error of the estimated difference between groups. df = 562.

S35 Table. Effect sizes for pairwise estimated marginal mean (EMM) comparisons between birth year groups for cephalic index (CI).

Contrast	Effect size	SE	Lower CL	Upper CL
birth_year2010 - birth_year2011	-0.32	0.17	-0.66	0.01
birth_year2010 - birth_year2012	-0.05	0.16	-0.36	0.27
birth_year2010 - birth_year2013	-0.32	0.24	-0.80	0.15
birth_year2010 - birth_year2014	-0.51	0.20	-0.89	-0.12
birth_year2010 - birth_year2015	-0.38	0.29	-0.95	0.20
birth_year2010 - birth_year2016	1.14	0.27	0.61	1.67
birth_year2010 - birth_year2017	0.59	0.20	0.20	0.98
birth_year2010 - birth_year2018	0.73	0.19	0.36	1.11
birth_year2010 - birth_year2019	0.51	0.20	0.12	0.91
birth_year2011 - birth_year2012	0.28	0.13	0.02	0.53
birth_year2011 - birth_year2013	-0.002	0.22	-0.44	0.44
birth_year2011 - birth_year2014	-0.18	0.17	-0.52	0.16
birth_year2011 - birth_year2015	-0.05	0.28	-0.60	0.49
birth_year2011 - birth_year2016	1.47	0.25	0.97	1.97
birth_year2011 - birth_year2017	0.91	0.17	0.57	1.26
birth_year2011 - birth_year2018	1.06	0.16	0.74	1.38
birth_year2011 - birth_year2019	0.83	0.18	0.48	1.18
birth_year2012 - birth_year2013	-0.28	0.22	-0.70	0.15
birth_year2012 - birth_year2014	-0.46	0.16	-0.78	-0.14
birth_year2012 - birth_year2015	-0.33	0.27	-0.86	0.20
birth_year2012 - birth_year2016	1.19	0.25	0.71	1.67
birth_year2012 - birth_year2017	0.64	0.16	0.32	0.96
birth_year2012 - birth_year2018	0.78	0.15	0.48	1.08
birth_year2012 - birth_year2019	0.56	0.17	0.23	0.89
birth_year2013 - birth_year2014	-0.18	0.24	-0.66	0.30
birth_year2013 - birth_year2015	-0.05	0.33	-0.69	0.59
birth_year2013 - birth_year2016	1.47	0.31	0.86	2.07
birth_year2013 - birth_year2017	0.92	0.25	0.43	1.40
birth_year2013 - birth_year2018	1.06	0.24	0.59	1.53
birth_year2013 - birth_year2019	0.84	0.25	0.35	1.32
birth_year2014 - birth_year2015	0.13	0.29	-0.45	0.71
birth_year2014 - birth_year2016	1.65	0.27	1.11	2.19
birth_year2014 - birth_year2017	1.10	0.20	0.70	1.49
birth_year2014 - birth_year2018	1.24	0.19	0.86	1.62
birth_year2014 - birth_year2019	1.02	0.21	0.61	1.42
birth_year2015 - birth_year2016	1.52	0.35	0.84	2.20
birth_year2015 - birth_year2017	0.97	0.30	0.39	1.55
birth_year2015 - birth_year2018	1.11	0.29	0.54	1.68
birth_year2015 - birth_year2019	0.89	0.30	0.30	1.47
birth_year2016 - birth_year2017	-0.55	0.27	-1.08	-0.02
birth_year2016 - birth_year2018	-0.41	0.26	-0.92	0.11
birth_year2016 - birth_year2019	-0.63	0.27	-1.17	-0.10
birth_year2017 - birth_year2018	0.14	0.19	-0.23	0.51
birth_year2017 - birth_year2019	-0.08	0.20	-0.48	0.31
birth_year2018 - birth_year2019	-0.22	0.19	-0.60	0.15

SE: standard error of the Effect size estimate. CL: Confidence level. df = 562.

Effect sizes are displayed to three decimal places if rounding up to two decimal places results in zero.

S36 Table. Estimated marginal mean (EMM) of globularity index (GI) for each birth year.

Birth year	EMM	SE	Lower CL	Upper CL
2010	0.986	0.001	0.983	0.988
2011	0.984	0.001	0.983	0.986
2012	0.983	0.001	0.981	0.984
2013	0.985	0.002	0.981	0.989
2014	0.984	0.001	0.982	0.987
2015	0.982	0.002	0.977	0.987
2016	0.978	0.002	0.973	0.982
2017	0.981	0.001	0.979	0.984
2018	0.981	0.001	0.979	0.983
2019	0.979	0.001	0.977	0.982

SE: Standard error of the EMM. CL: Confidence limit. df = 562

Values are displayed to three decimal places as rounding up to two decimal places either results in zero or the same value across most entries.

S37 Table. Pairwise comparisons of estimated marginal means (EMM) between birth year groups for globularity index (GI).

Contrast	EMM difference	SE	t.ratio	P value	Significance
birth_year2010 - birth_year2011	0.001	0.002	0.73	0.60	ns
birth_year2010 - birth_year2012	0.003	0.002	1.89	0.17	ns
birth_year2010 - birth_year2013	0.001	0.002	0.30	0.85	ns
birth_year2010 - birth_year2014	0.001	0.002	0.69	0.60	ns
birth_year2010 - birth_year2015	0.004	0.003	1.31	0.37	ns
birth_year2010 - birth_year2016	0.008	0.003	3.10	0.045	*
birth_year2010 - birth_year2017	0.004	0.002	2.35	0.09	ns
birth_year2010 - birth_year2018	0.005	0.002	2.62	0.07	ns
birth_year2010 - birth_year2019	0.006	0.002	3.21	0.045	*
birth_year2011 - birth_year2012	0.002	0.001	1.38	0.37	ns
birth_year2011 - birth_year2013	4.84×10^{-4}	0.002	-0.23	0.86	ns
birth_year2011 - birth_year2014	1.11×10^{-4}	0.002	0.07	0.95	ns
birth_year2011 - birth_year2015	0.002	0.003	0.93	0.53	ns
birth_year2011 - birth_year2016	0.007	0.002	2.82	0.06	ns
birth_year2011 - birth_year2017	0.003	0.002	1.97	0.16	ns
birth_year2011 - birth_year2018	0.003	0.002	2.27	0.10	ns
birth_year2011 - birth_year2019	0.005	0.002	2.95	0.04998	*
birth_year2012 - birth_year2013	-0.002	0.002	-1.06	0.50	ns
birth_year2012 - birth_year2014	-0.002	0.002	-1.03	0.50	ns
birth_year2012 - birth_year2015	0.001	0.003	0.29	0.85	ns
birth_year2012 - birth_year2016	0.005	0.002	2.16	0.12	ns
birth_year2012 - birth_year2017	0.002	0.002	0.98	0.52	ns
birth_year2012 - birth_year2018	0.002	0.001	1.24	0.39	ns
birth_year2012 - birth_year2019	0.003	0.002	2.04	0.15	ns
birth_year2013 - birth_year2014	0.001	0.002	0.26	0.85	ns
birth_year2013 - birth_year2015	0.003	0.003	0.95	0.53	ns
birth_year2013 - birth_year2016	0.007	0.003	2.49	0.07	ns
birth_year2013 - birth_year2017	0.004	0.002	1.60	0.26	ns
birth_year2013 - birth_year2018	0.004	0.002	1.76	0.21	ns
birth_year2013 - birth_year2019	0.005	0.002	2.31	0.10	ns
birth_year2014 - birth_year2015	0.002	0.003	0.84	0.55	ns
birth_year2014 - birth_year2016	0.007	0.003	2.58	0.07	ns
birth_year2014 - birth_year2017	0.003	0.002	1.65	0.25	ns
birth_year2014 - birth_year2018	0.003	0.002	1.88	0.17	ns
birth_year2014 - birth_year2019	0.005	0.002	2.50	0.07	ns
birth_year2015 - birth_year2016	0.004	0.003	1.30	0.37	ns
birth_year2015 - birth_year2017	0.001	0.003	0.27	0.85	ns
birth_year2015 - birth_year2018	0.001	0.003	0.37	0.84	ns
birth_year2015 - birth_year2019	0.002	0.003	0.88	0.53	ns
birth_year2016 - birth_year2017	-0.003	0.003	-1.37	0.37	ns
birth_year2016 - birth_year2018	-0.003	0.002	-1.30	0.37	ns
birth_year2016 - birth_year2019	-0.002	0.003	-0.69	0.60	ns
birth_year2017 - birth_year2018	2.50×10^{-4}	0.002	0.14	0.91	ns
birth_year2017 - birth_year2019	0.002	0.002	0.90	0.53	ns
birth_year2018 - birth_year2019	0.001	0.002	0.80	0.56	ns

SE: standard error of the estimated difference between groups. df = 562.

EMM values are displayed to three decimal places or three significant figures as rounding up to two decimal places either results in zero or the same value across most entries.

S38 Table. Effect sizes for pairwise estimated marginal mean (EMM) comparisons between birth year groups for globularity index (GI).

Contrast	Effect size	SE	Lower CL	Upper CL
birth_year2010 - birth_year2011	0.13	0.17	-0.21	0.46
birth_year2010 - birth_year2012	0.30	0.16	-0.01	0.62
birth_year2010 - birth_year2013	0.07	0.24	-0.40	0.55
birth_year2010 - birth_year2014	0.14	0.20	-0.25	0.52
birth_year2010 - birth_year2015	0.38	0.29	-0.19	0.96
birth_year2010 - birth_year2016	0.83	0.27	0.30	1.36
birth_year2010 - birth_year2017	0.46	0.20	0.08	0.85
birth_year2010 - birth_year2018	0.49	0.19	0.12	0.86
birth_year2010 - birth_year2019	0.64	0.20	0.25	1.04
birth_year2011 - birth_year2012	0.18	0.13	-0.08	0.43
birth_year2011 - birth_year2013	-0.05	0.22	-0.49	0.39
birth_year2011 - birth_year2014	0.01	0.17	-0.33	0.35
birth_year2011 - birth_year2015	0.26	0.28	-0.29	0.80
birth_year2011 - birth_year2016	0.71	0.25	0.21	1.20
birth_year2011 - birth_year2017	0.34	0.17	0.00	0.68
birth_year2011 - birth_year2018	0.37	0.16	0.05	0.68
birth_year2011 - birth_year2019	0.52	0.18	0.17	0.87
birth_year2012 - birth_year2013	-0.23	0.22	-0.66	0.19
birth_year2012 - birth_year2014	-0.17	0.16	-0.49	0.15
birth_year2012 - birth_year2015	0.08	0.27	-0.45	0.61
birth_year2012 - birth_year2016	0.53	0.24	0.05	1.01
birth_year2012 - birth_year2017	0.16	0.16	-0.16	0.48
birth_year2012 - birth_year2018	0.19	0.15	-0.11	0.48
birth_year2012 - birth_year2019	0.34	0.17	0.01	0.67
birth_year2013 - birth_year2014	0.06	0.24	-0.42	0.54
birth_year2013 - birth_year2015	0.31	0.33	-0.33	0.95
birth_year2013 - birth_year2016	0.76	0.31	0.16	1.36
birth_year2013 - birth_year2017	0.39	0.24	-0.09	0.87
birth_year2013 - birth_year2018	0.42	0.24	-0.05	0.88
birth_year2013 - birth_year2019	0.57	0.25	0.08	1.06
birth_year2014 - birth_year2015	0.25	0.29	-0.33	0.82
birth_year2014 - birth_year2016	0.69	0.27	0.16	1.22
birth_year2014 - birth_year2017	0.33	0.20	-0.06	0.72
birth_year2014 - birth_year2018	0.35	0.19	-0.02	0.72
birth_year2014 - birth_year2019	0.51	0.20	0.11	0.91
birth_year2015 - birth_year2016	0.45	0.35	-0.23	1.13
birth_year2015 - birth_year2017	0.08	0.29	-0.50	0.66
birth_year2015 - birth_year2018	0.11	0.29	-0.46	0.67
birth_year2015 - birth_year2019	0.26	0.30	-0.32	0.84
birth_year2016 - birth_year2017	-0.37	0.27	-0.90	0.16
birth_year2016 - birth_year2018	-0.34	0.26	-0.86	0.17
birth_year2016 - birth_year2019	-0.19	0.27	-0.72	0.35
birth_year2017 - birth_year2018	0.03	0.19	-0.34	0.40
birth_year2017 - birth_year2019	0.18	0.20	-0.22	0.58
birth_year2018 - birth_year2019	0.15	0.19	-0.22	0.53

SE: standard error of the effect size estimate. CL: Confidence level. df = 562.

S39 Table. Estimated marginal mean (EMM) of volume for each birth year.

Birth year	EMM	SE	Lower CL	Upper CL
2010	744.96	8.95	727.38	762.54
2011	752.56	6.45	739.88	765.23
2012	759.48	5.31	749.05	769.91
2013	756.67	12.91	731.32	782.02
2014	749.36	9.04	731.61	767.11
2015	731.07	16.66	698.35	763.80
2016	710.94	14.80	681.86	740.02
2017	746.48	9.04	728.73	764.23
2018	747.22	8.13	731.25	763.19
2019	755.36	9.36	736.98	773.74

SE: Standard error of the EMM. CL: Confidence limit. df = 562

S40 Table. Pairwise comparisons of estimated marginal means (EMM) between birth year groups for volume.

Contrast	EMM difference	SE	t ratio	P value	Significance
birth_year2010 - birth_year2011	-7.60	11.03	-0.69	0.83	ns
birth_year2010 - birth_year2012	-14.52	10.40	-1.40	0.69	ns
birth_year2010 - birth_year2013	-11.71	15.70	-0.75	0.83	ns
birth_year2010 - birth_year2014	-4.40	12.71	-0.35	0.92	ns
birth_year2010 - birth_year2015	13.89	18.91	0.73	0.83	ns
birth_year2010 - birth_year2016	34.02	17.30	1.97	0.28	ns
birth_year2010 - birth_year2017	-1.52	12.72	-0.12	0.95	ns
birth_year2010 - birth_year2018	-2.26	12.09	-0.19	0.92	ns
birth_year2010 - birth_year2019	-10.40	12.96	-0.80	0.83	ns
birth_year2011 - birth_year2012	-6.92	8.35	-0.83	0.83	ns
birth_year2011 - birth_year2013	-4.11	14.43	-0.28	0.92	ns
birth_year2011 - birth_year2014	3.20	11.09	0.29	0.92	ns
birth_year2011 - birth_year2015	21.48	17.87	1.20	0.69	ns
birth_year2011 - birth_year2016	41.61	16.15	2.58	0.17	ns
birth_year2011 - birth_year2017	6.07	11.11	0.55	0.88	ns
birth_year2011 - birth_year2018	5.33	10.38	0.51	0.88	ns
birth_year2011 - birth_year2019	-2.81	11.38	-0.25	0.92	ns
birth_year2012 - birth_year2013	2.81	13.96	0.20	0.92	ns
birth_year2012 - birth_year2014	10.12	10.45	0.97	0.83	ns
birth_year2012 - birth_year2015	28.41	17.48	1.62	0.52	ns
birth_year2012 - birth_year2016	48.54	15.73	3.09	0.10	ns
birth_year2012 - birth_year2017	13.00	10.49	1.24	0.69	ns
birth_year2012 - birth_year2018	12.26	9.71	1.26	0.69	ns
birth_year2012 - birth_year2019	4.12	10.78	0.38	0.92	ns
birth_year2013 - birth_year2014	7.31	15.76	0.46	0.88	ns
birth_year2013 - birth_year2015	25.59	21.08	1.21	0.69	ns
birth_year2013 - birth_year2016	45.73	19.64	2.33	0.23	ns
birth_year2013 - birth_year2017	10.19	15.75	0.65	0.83	ns
birth_year2013 - birth_year2018	9.45	15.25	0.62	0.83	ns
birth_year2013 - birth_year2019	1.31	15.93	0.08	0.95	ns
birth_year2014 - birth_year2015	18.29	18.94	0.97	0.83	ns
birth_year2014 - birth_year2016	38.42	17.35	2.21	0.24	ns
birth_year2014 - birth_year2017	2.88	12.80	0.22	0.92	ns
birth_year2014 - birth_year2018	2.14	12.16	0.18	0.92	ns
birth_year2014 - birth_year2019	-6.00	13.10	-0.46	0.88	ns
birth_year2015 - birth_year2016	20.13	22.29	0.90	0.83	ns
birth_year2015 - birth_year2017	-15.41	18.96	-0.81	0.83	ns
birth_year2015 - birth_year2018	-16.15	18.54	-0.87	0.83	ns
birth_year2015 - birth_year2019	-24.29	19.12	-1.27	0.69	ns
birth_year2016 - birth_year2017	-35.54	17.34	-2.05	0.26	ns
birth_year2016 - birth_year2018	-36.28	16.89	-2.15	0.24	ns
birth_year2016 - birth_year2019	-44.42	17.51	-2.54	0.17	ns
birth_year2017 - birth_year2018	-0.74	12.15	-0.06	0.95	ns
birth_year2017 - birth_year2019	-8.88	13.00	-0.68	0.83	ns
birth_year2018 - birth_year2019	-8.14	12.39	-0.66	0.83	ns

SE: standard error of the estimated difference between groups. df = 562.

S41 Table. Effect sizes for pairwise estimated marginal mean (EMM) comparisons between birth year groups for volume.

Contrast	Effect size	SE	Lower CL	Upper CL
birth_year2010 - birth_year2011	-0.12	0.17	-0.45	0.22
birth_year2010 - birth_year2012	-0.23	0.16	-0.54	0.09
birth_year2010 - birth_year2013	-0.18	0.24	-0.66	0.30
birth_year2010 - birth_year2014	-0.07	0.20	-0.46	0.32
birth_year2010 - birth_year2015	0.22	0.29	-0.36	0.79
birth_year2010 - birth_year2016	0.53	0.27	-2.7×10^{-4}	1.05
birth_year2010 - birth_year2017	-0.02	0.20	-0.41	0.36
birth_year2010 - birth_year2018	-0.04	0.19	-0.40	0.33
birth_year2010 - birth_year2019	-0.16	0.20	-0.56	0.23
birth_year2011 - birth_year2012	-0.11	0.13	-0.36	0.15
birth_year2011 - birth_year2013	-0.06	0.22	-0.50	0.38
birth_year2011 - birth_year2014	0.05	0.17	-0.29	0.39
birth_year2011 - birth_year2015	0.33	0.28	-0.21	0.88
birth_year2011 - birth_year2016	0.64	0.25	0.15	1.14
birth_year2011 - birth_year2017	0.09	0.17	-0.24	0.43
birth_year2011 - birth_year2018	0.08	0.16	-0.23	0.40
birth_year2011 - birth_year2019	-0.04	0.18	-0.39	0.30
birth_year2012 - birth_year2013	0.04	0.22	-0.38	0.47
birth_year2012 - birth_year2014	0.16	0.16	-0.16	0.48
birth_year2012 - birth_year2015	0.44	0.27	-0.09	0.97
birth_year2012 - birth_year2016	0.75	0.24	0.27	1.23
birth_year2012 - birth_year2017	0.20	0.16	-0.12	0.52
birth_year2012 - birth_year2018	0.19	0.15	-0.11	0.49
birth_year2012 - birth_year2019	0.06	0.17	-0.26	0.39
birth_year2013 - birth_year2014	0.11	0.24	-0.37	0.59
birth_year2013 - birth_year2015	0.40	0.33	-0.25	1.04
birth_year2013 - birth_year2016	0.71	0.31	0.11	1.31
birth_year2013 - birth_year2017	0.16	0.24	-0.32	0.64
birth_year2013 - birth_year2018	0.15	0.24	-0.32	0.61
birth_year2013 - birth_year2019	0.02	0.25	-0.46	0.51
birth_year2014 - birth_year2015	0.28	0.29	-0.29	0.86
birth_year2014 - birth_year2016	0.60	0.27	0.07	1.12
birth_year2014 - birth_year2017	0.04	0.20	-0.34	0.43
birth_year2014 - birth_year2018	0.03	0.19	-0.34	0.40
birth_year2014 - birth_year2019	-0.09	0.20	-0.49	0.31
birth_year2015 - birth_year2016	0.31	0.35	-0.37	0.99
birth_year2015 - birth_year2017	-0.24	0.29	-0.82	0.34
birth_year2015 - birth_year2018	-0.25	0.29	-0.81	0.31
birth_year2015 - birth_year2019	-0.38	0.30	-0.96	0.21
birth_year2016 - birth_year2017	-0.55	0.27	-1.08	-0.02
birth_year2016 - birth_year2018	-0.56	0.26	-1.08	-0.05
birth_year2016 - birth_year2019	-0.69	0.27	-1.22	-0.15
birth_year2017 - birth_year2018	-0.01	0.19	-0.38	0.36
birth_year2017 - birth_year2019	-0.14	0.20	-0.53	0.26
birth_year2018 - birth_year2019	-0.13	0.19	-0.50	0.25

SE: standard error of the effect size estimate. CL: Confidence level. df = 562.

S42 Table. Estimated marginal mean (EMM) of height-half length ratio (h/a_2) for each birth year.

Birth year	EMM	SE	Lower CL	Upper CL
2010	1.19	0.01	1.16	1.21
2011	1.27	0.01	1.25	1.29
2012	1.26	0.01	1.24	1.27
2013	1.28	0.02	1.24	1.32
2014	1.29	0.01	1.26	1.31
2015	1.24	0.02	1.20	1.29
2016	1.09	0.02	1.05	1.13
2017	1.18	0.01	1.15	1.20
2018	1.17	0.01	1.14	1.19
2019	1.21	0.01	1.18	1.23

SE: Standard error of the EMM. CL: Confidence limit. df = 562

S43 Table. Pairwise comparisons of estimated marginal means (EMM) between birth year groups for height-half length ratio (*h/a_2*).

Contrast	EMM difference	SE	t ratio	P value	Significance
birth_year2010 - birth_year2011	-0.08	0.02	-5.08	< 0.001	***
birth_year2010 - birth_year2012	-0.07	0.02	-4.47	< 0.001	***
birth_year2010 - birth_year2013	-0.09	0.02	-3.97	< 0.001	***
birth_year2010 - birth_year2014	-0.10	0.02	-5.38	< 0.001	***
birth_year2010 - birth_year2015	-0.06	0.03	-2.07	0.06	ns
birth_year2010 - birth_year2016	0.09	0.03	3.75	< 0.001	***
birth_year2010 - birth_year2017	0.01	0.02	0.55	0.63	ns
birth_year2010 - birth_year2018	0.02	0.02	1.13	0.32	ns
birth_year2010 - birth_year2019	-0.02	0.02	-1.06	0.34	ns
birth_year2011 - birth_year2012	0.01	0.01	1.14	0.32	ns
birth_year2011 - birth_year2013	-0.01	0.02	-0.44	0.69	ns
birth_year2011 - birth_year2014	-0.02	0.02	-1.12	0.32	ns
birth_year2011 - birth_year2015	0.02	0.03	0.94	0.39	ns
birth_year2011 - birth_year2016	0.18	0.02	7.49	< 0.001	***
birth_year2011 - birth_year2017	0.09	0.02	5.67	< 0.001	***
birth_year2011 - birth_year2018	0.10	0.02	6.71	< 0.001	***
birth_year2011 - birth_year2019	0.06	0.02	3.72	< 0.001	***
birth_year2012 - birth_year2013	-0.02	0.02	-1.14	0.32	ns
birth_year2012 - birth_year2014	-0.03	0.02	-2.10	0.06	ns
birth_year2012 - birth_year2015	0.01	0.03	0.41	0.70	ns
birth_year2012 - birth_year2016	0.16	0.02	7.08	< 0.001	***
birth_year2012 - birth_year2017	0.08	0.02	5.10	< 0.001	***
birth_year2012 - birth_year2018	0.09	0.01	6.18	< 0.001	***
birth_year2012 - birth_year2019	0.05	0.02	3.04	0.005	**
birth_year2013 - birth_year2014	-0.01	0.02	-0.39	0.70	ns
birth_year2013 - birth_year2015	0.03	0.03	1.10	0.32	ns
birth_year2013 - birth_year2016	0.19	0.03	6.48	< 0.001	***
birth_year2013 - birth_year2017	0.10	0.02	4.40	< 0.001	***
birth_year2013 - birth_year2018	0.11	0.02	4.98	< 0.001	***
birth_year2013 - birth_year2019	0.07	0.02	3.05	0.005	**
birth_year2014 - birth_year2015	0.04	0.03	1.54	0.17	ns
birth_year2014 - birth_year2016	0.19	0.03	7.68	< 0.001	***
birth_year2014 - birth_year2017	0.11	0.02	5.89	< 0.001	***
birth_year2014 - birth_year2018	0.12	0.02	6.74	< 0.001	***
birth_year2014 - birth_year2019	0.08	0.02	4.18	< 0.001	***
birth_year2015 - birth_year2016	0.15	0.03	4.67	< 0.001	***
birth_year2015 - birth_year2017	0.07	0.03	2.44	0.03	*
birth_year2015 - birth_year2018	0.08	0.03	2.85	0.01	**
birth_year2015 - birth_year2019	0.04	0.03	1.34	0.25	ns
birth_year2016 - birth_year2017	-0.08	0.03	-3.34	0.002	**
birth_year2016 - birth_year2018	-0.08	0.02	-3.04	0.005	**
birth_year2016 - birth_year2019	-0.11	0.03	-4.49	< 0.001	***
birth_year2017 - birth_year2018	0.01	0.02	0.54	0.63	ns
birth_year2017 - birth_year2019	-0.03	0.02	-1.59	0.16	ns
birth_year2018 - birth_year2019	-0.04	0.02	-2.20	0.04	*

SE: standard error of the estimated difference between groups. df = 562.

S44 Table. Effect sizes for pairwise estimated marginal mean (EMM) comparisons between birth year groups for height-half length ratio (h/a_2).

Contrast	Effect size	SE	Lower CL	Upper CL
birth_year2010 - birth_year2011	-0.87	0.17	-1.21	-0.53
birth_year2010 - birth_year2012	-0.72	0.16	-1.04	-0.40
birth_year2010 - birth_year2013	-0.97	0.25	-1.45	-0.48
birth_year2010 - birth_year2014	-1.06	0.20	-1.45	-0.67
birth_year2010 - birth_year2015	-0.61	0.29	-1.18	-0.03
birth_year2010 - birth_year2016	1.01	0.27	0.48	1.54
birth_year2010 - birth_year2017	0.11	0.20	-0.28	0.50
birth_year2010 - birth_year2018	0.21	0.19	-0.16	0.58
birth_year2010 - birth_year2019	-0.21	0.20	-0.61	0.18
birth_year2011 - birth_year2012	0.15	0.13	-0.11	0.40
birth_year2011 - birth_year2013	-0.10	0.22	-0.54	0.34
birth_year2011 - birth_year2014	-0.19	0.17	-0.53	0.15
birth_year2011 - birth_year2015	0.26	0.28	-0.28	0.80
birth_year2011 - birth_year2016	1.87	0.26	1.37	2.38
birth_year2011 - birth_year2017	0.98	0.17	0.63	1.32
birth_year2011 - birth_year2018	1.08	0.16	0.76	1.40
birth_year2011 - birth_year2019	0.66	0.18	0.31	1.00
birth_year2012 - birth_year2013	-0.25	0.22	-0.67	0.18
birth_year2012 - birth_year2014	-0.34	0.16	-0.66	-0.02
birth_year2012 - birth_year2015	0.11	0.27	-0.42	0.64
birth_year2012 - birth_year2016	1.73	0.25	1.24	2.22
birth_year2012 - birth_year2017	0.83	0.16	0.51	1.15
birth_year2012 - birth_year2018	0.93	0.15	0.63	1.23
birth_year2012 - birth_year2019	0.51	0.17	0.18	0.84
birth_year2013 - birth_year2014	-0.09	0.24	-0.57	0.39
birth_year2013 - birth_year2015	0.36	0.33	-0.28	1.00
birth_year2013 - birth_year2016	1.97	0.31	1.36	2.58
birth_year2013 - birth_year2017	1.07	0.25	0.59	1.56
birth_year2013 - birth_year2018	1.18	0.24	0.71	1.65
birth_year2013 - birth_year2019	0.75	0.25	0.27	1.24
birth_year2014 - birth_year2015	0.45	0.29	-0.13	1.03
birth_year2014 - birth_year2016	2.07	0.28	1.52	2.61
birth_year2014 - birth_year2017	1.17	0.20	0.77	1.56
birth_year2014 - birth_year2018	1.27	0.19	0.89	1.65
birth_year2014 - birth_year2019	0.85	0.20	0.45	1.25
birth_year2015 - birth_year2016	1.61	0.35	0.93	2.30
birth_year2015 - birth_year2017	0.72	0.29	0.14	1.29
birth_year2015 - birth_year2018	0.82	0.29	0.25	1.39
birth_year2015 - birth_year2019	0.40	0.30	-0.19	0.98
birth_year2016 - birth_year2017	-0.90	0.27	-1.43	-0.37
birth_year2016 - birth_year2018	-0.80	0.26	-1.31	-0.28
birth_year2016 - birth_year2019	-1.22	0.27	-1.76	-0.68
birth_year2017 - birth_year2018	0.10	0.19	-0.27	0.47
birth_year2017 - birth_year2019	-0.32	0.20	-0.72	0.08
birth_year2018 - birth_year2019	-0.42	0.19	-0.80	-0.05

SE: standard error of the Effect size estimate. CL: Confidence level. df = 562.
