# Supplementary material for: Changes in infant head shape: Developmental trends during the first year of life and secular changes observed in recent years
Source: PLoS One. 2026 Mar 13;21(3):e0344700. doi: 10.1371/journal.pone.0344700 (PMC12987498; doi:10.1371/journal.pone.0344700)
Supplement: S2 File — (PDF) [file pone.0344700.s002.pdf]

Supplementary Figures

Title

Changes in Infant Head Shape: Developmental Trends During the First Year of Life and Secular Changes Observed in Recent Years

Short title

Developmental and Secular Changes in the Infant Head Shape

Eujin Lee[1], Hama Watanabe[1], Ryoya Saji[2,3], Fumitaka Homae[4,5] Gentaro Taga[1]

[1] Graduate School of Education, The University of Tokyo, Tokyo, Japan

[2] College of Agriculture, Tamagawa University, Tokyo, Japan

[3] Brain Science Institute, Tamagawa University, Tokyo, Japan

[4] Department of Language Sciences, Tokyo Metropolitan University, Tokyo, Japan

[5] Research Center for Language, Brain and Genetics, Tokyo Metropolitan University, Tokyo, Japan

Corresponding author: Eujin Lee

Email: elee715@p.u-tokyo.ac.jp

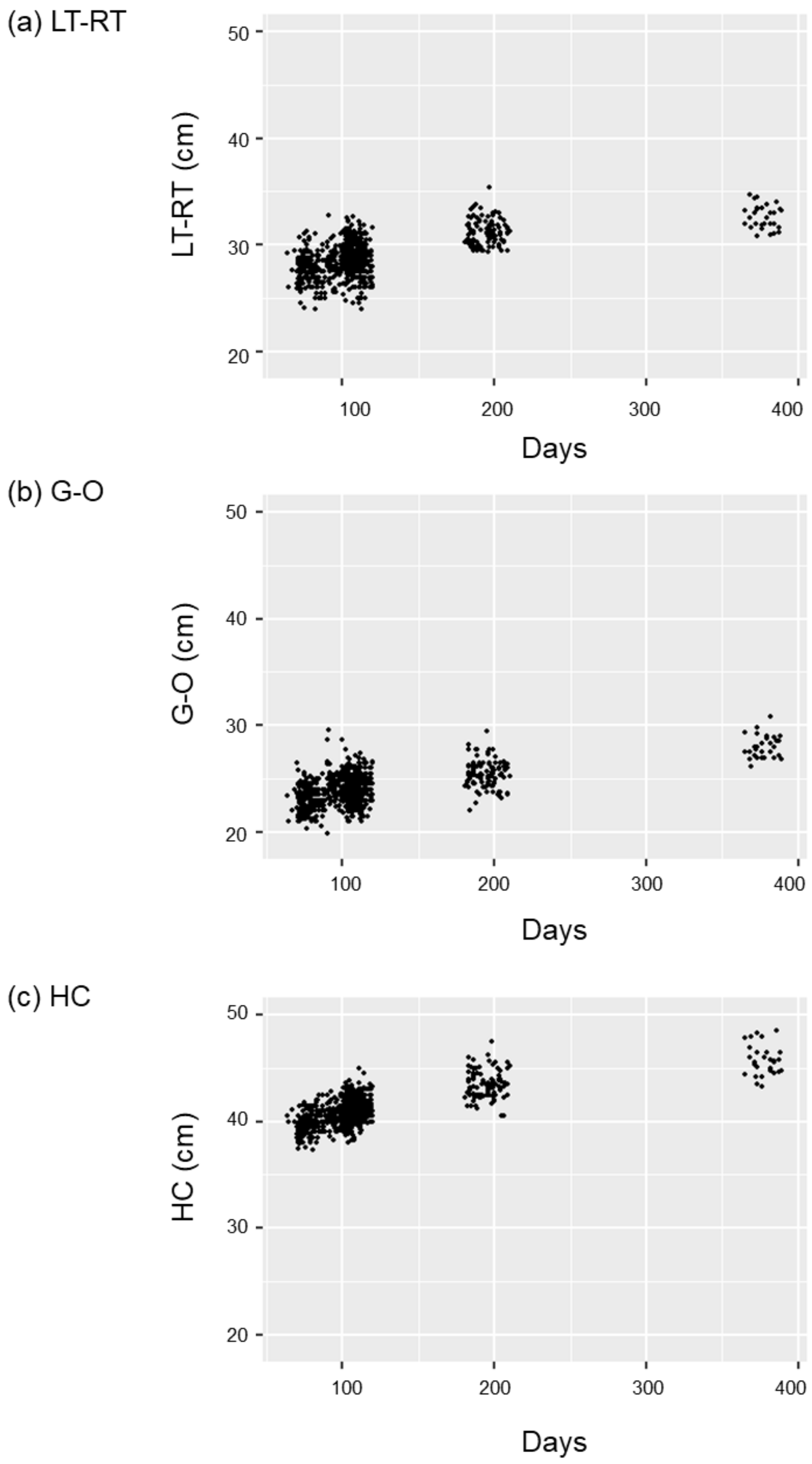

S1 Fig. Scatter plots of the direct measurements (left-tragion-right-tragion (LT-RT), glabella-occipital protuberance (G-O), and head circumference (HC)).

(a) Left tragion-right tragion (LT-RT), (b) glabella-occipital protuberance (G-O), and (c) head circumference (HC). Infants aged 2 months (n = 207), 3 months (n =578), 6 months (n = 99), and 12 months (n = 29) with all three direct measurements are represented.

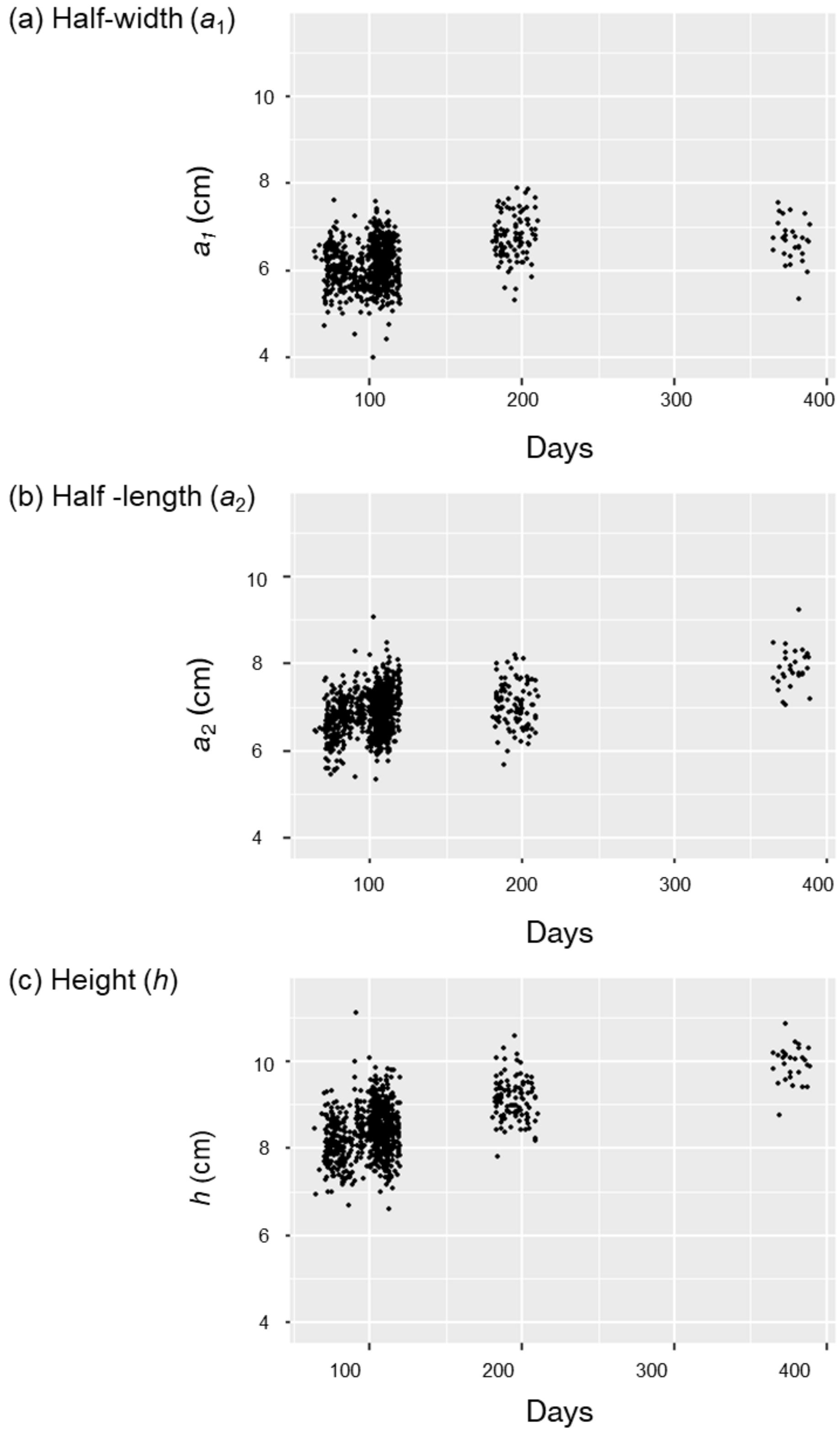

S2 Fig. Scatter plots of the estimated measurements (half-width (a_1), half-length (a_2), and height (h)).

(a) Half-width (a_1), (b) half-length (a_2), and (c) height (h). Infants aged 2 months (n = 207), 3 months (n =578), 6 months (n = 99), and 12 months (n = 29) with estimated measurements derived from the three direct measurements (left tragion-right tragion (LT-RT), glabella-occipital protuberance (G-O), and head circumference (HC)) are represented.

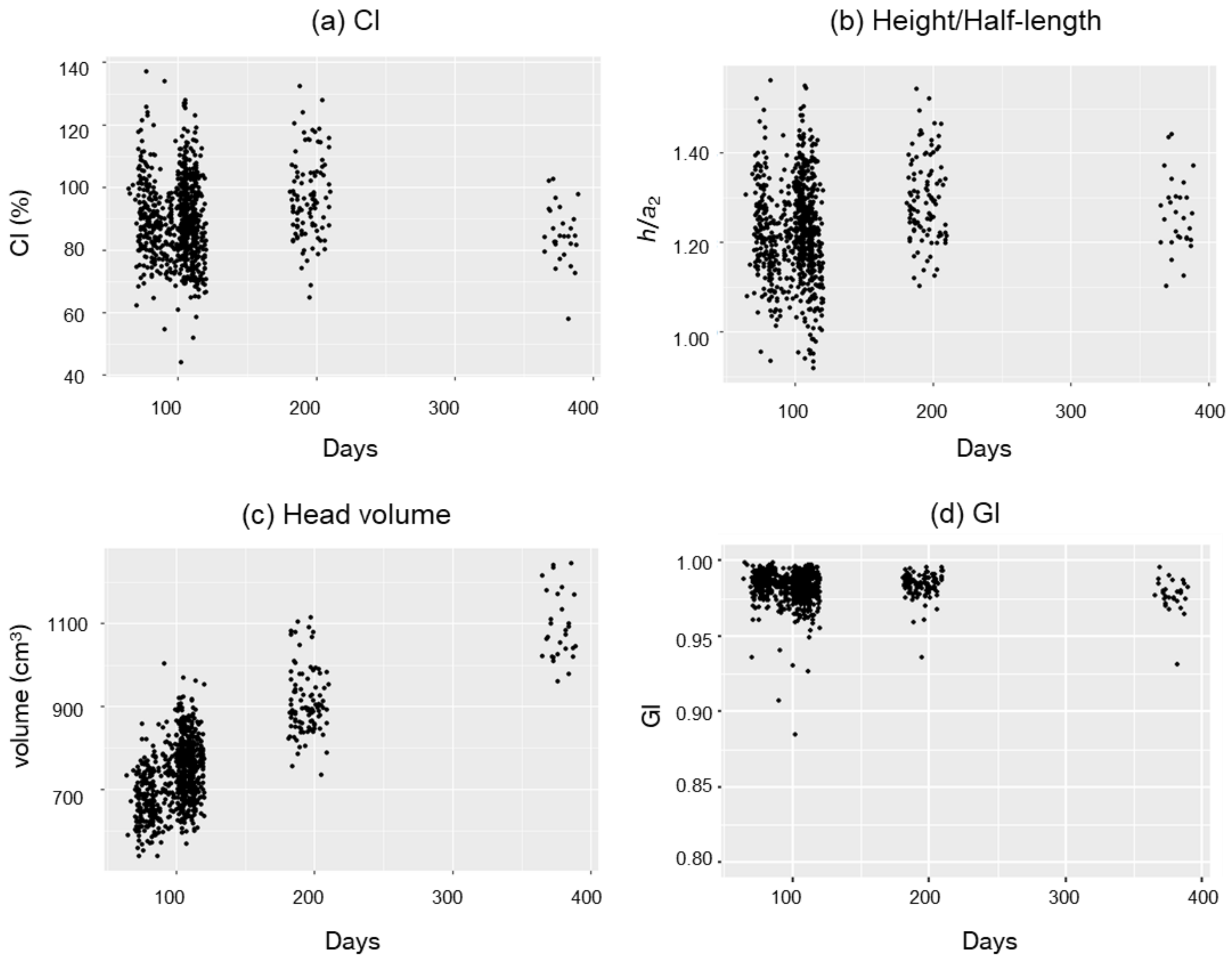

S3 Fig. Scatter plots of the estimated measurements (Cephalic Index (CI), height/half-length ratio, head volume and Globularity Index (GI)).

(a) Cephalic Index (CI), (b) height/half-length ratio, (c) head volume and (d) Globularity Index (GI). Infants aged 2 months (n = 207), 3 months (n =578), 6 months (n = 99), and 12 months (n = 29) with CI, GI, height/half-length ratio and head volume measurements derived from the three direct measurements (left tragion-right tragion (LT-RT), glabella-occipital protuberance (G-O), and head circumference (HC)) are represented.

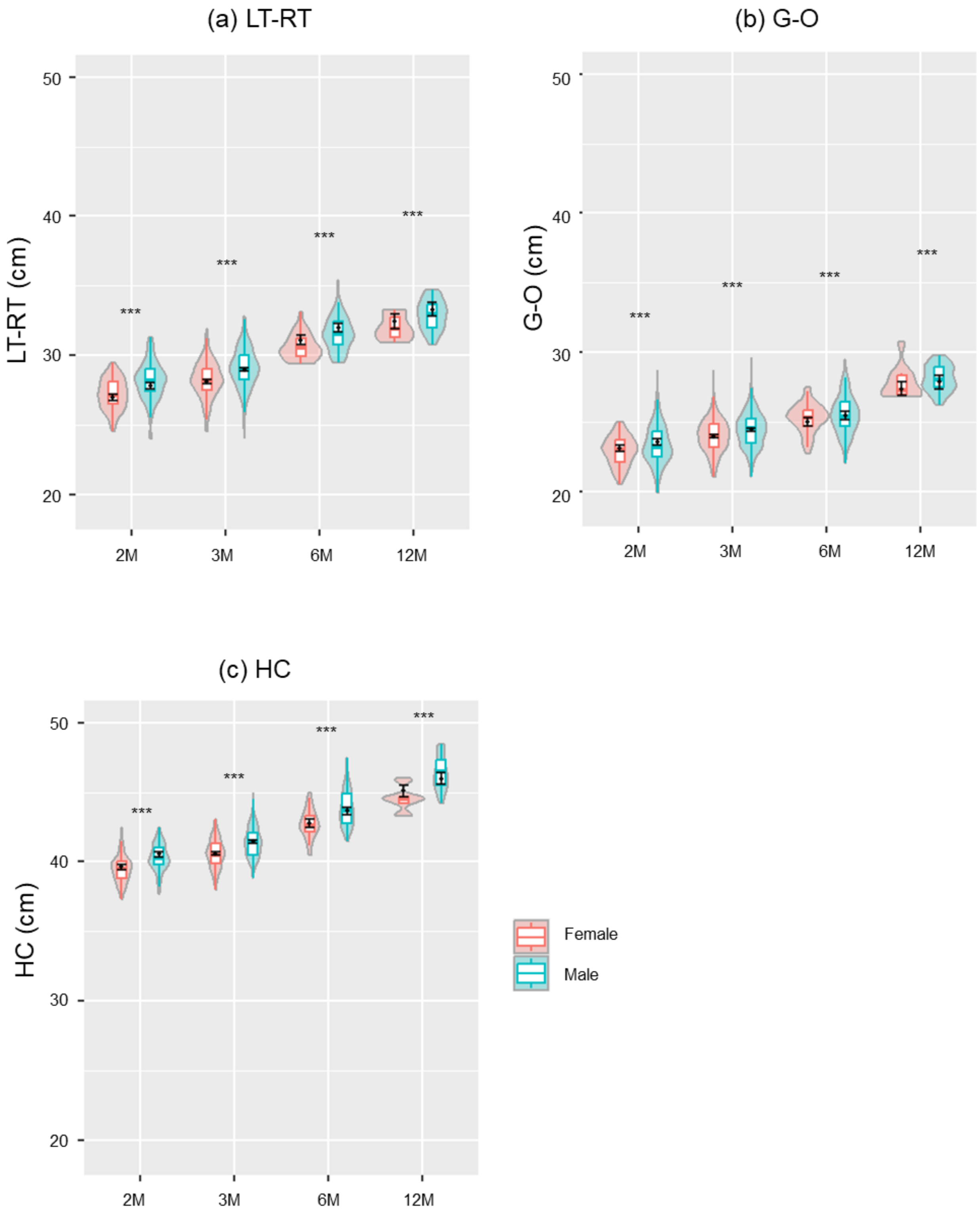

S4 Fig. Sex differences in the direct (left tragion-right tragion (LT-RT), glabella-occipital protuberance (G-O) and head circumference (HC)) measurements observed during the first 12 months after birth.
(a) LT-RT, (b) G-O, (c) HC. Statistical differences between sexes are indicated as follows: ***($p < 0.001$).

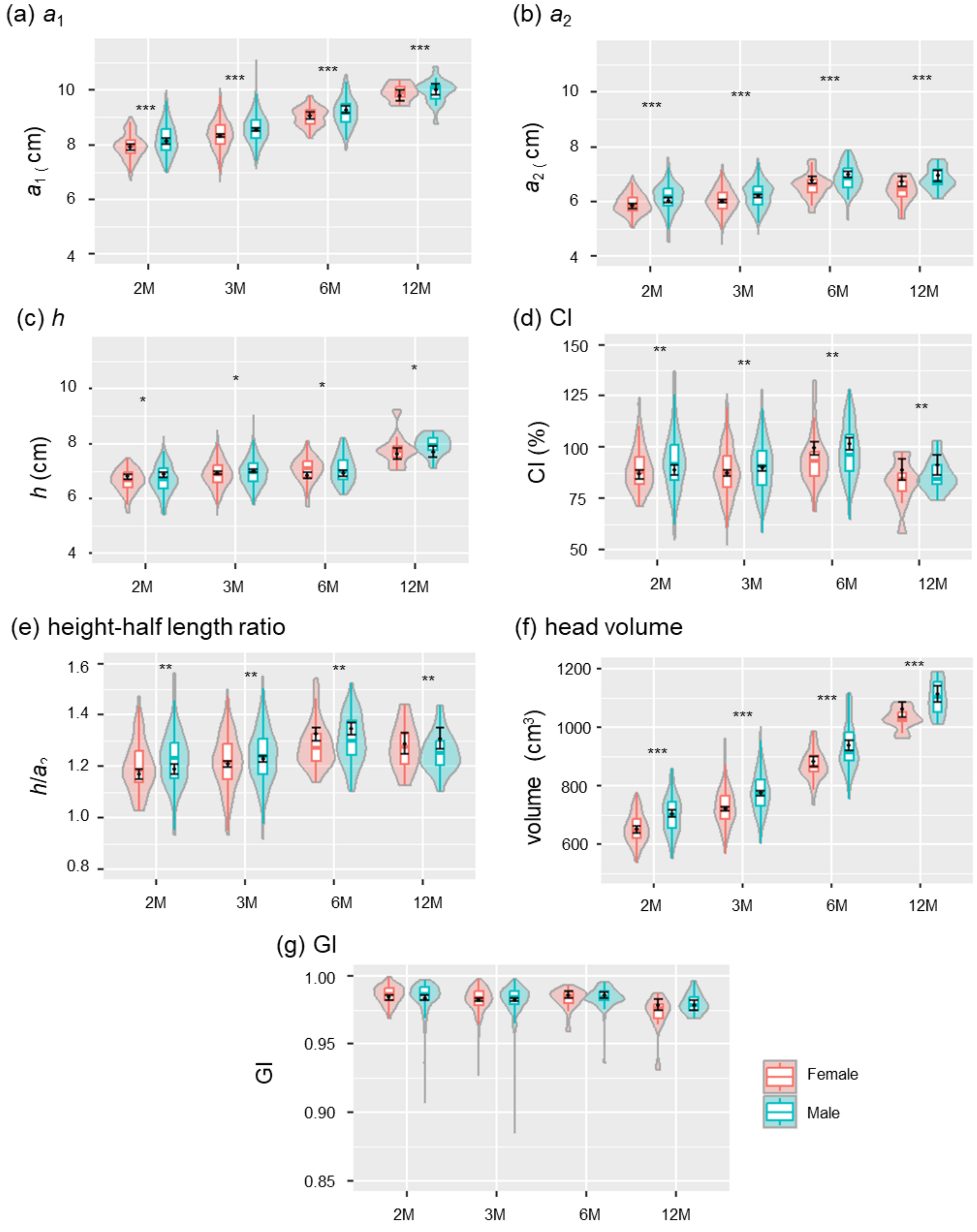

S5 Fig. Sex differences in the estimated (half-width (a_1), half-length (a_2), height (h), CI,height/half-length (h/a_2), head volume and GI) measurements observed during the first 12 months after birth.

(a) h, (b) a_1, (c) a_2, (d) CI, (e) height/half-length (h/a_2), (f) head volume and (g) GI. Statistical differences between sexes are indicated as follows: *($p < 0.05$), **($p < 0.01$), ***($p < 0.001$) and none (not significant).

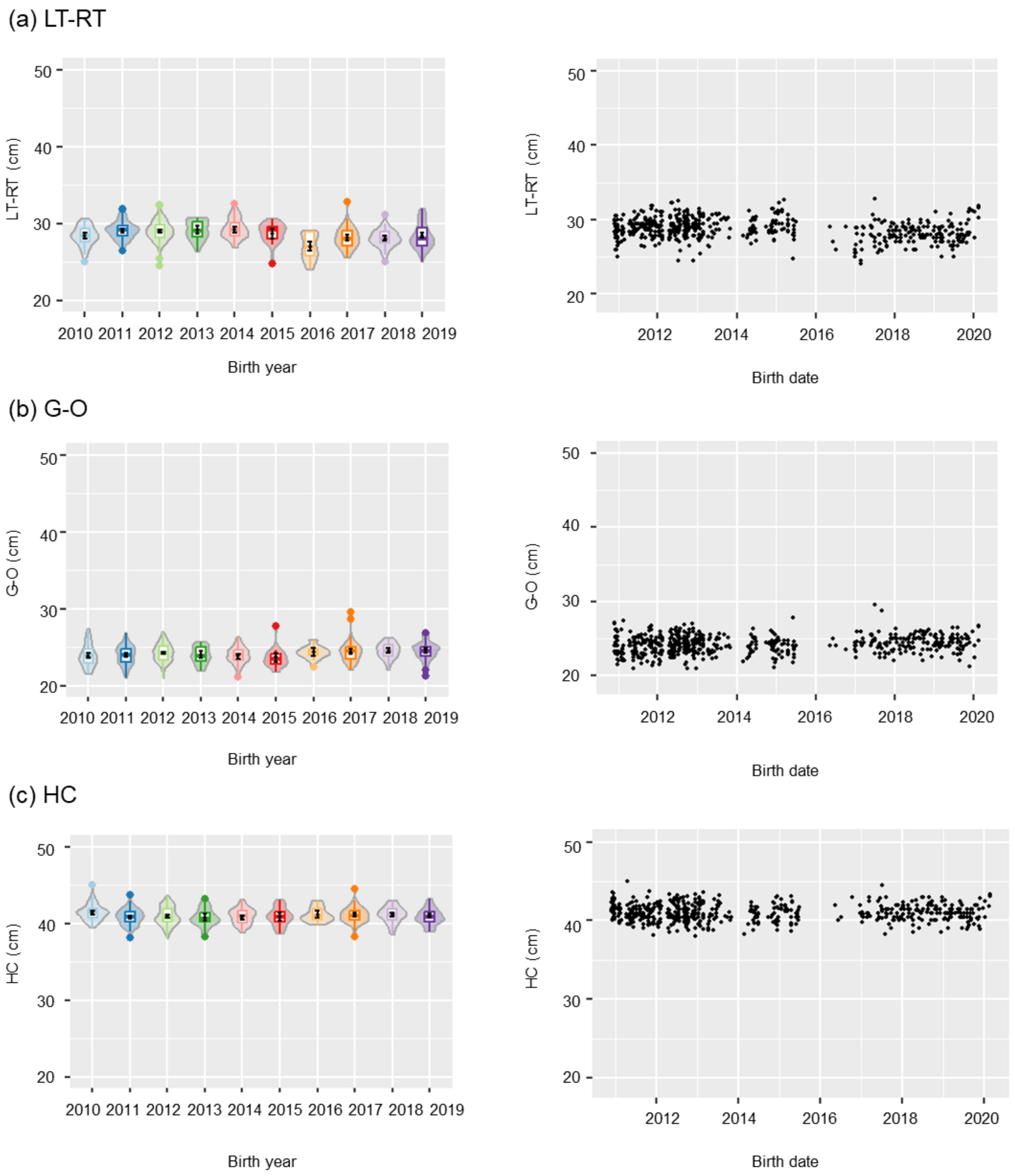

S6 Fig. Boxplots and scatter plots displaying trends observed in the direct measurements (left tragion-right tragion (LT-RT), glabella-occipital protuberance (G-O), and head circumference (HC)) at 3 months during 2010-2019.

(a) Left tragion-right tragion (LT-RT), (b) glabella-occipital protuberance (G-O), and (c) head circumference (HC). Infants aged 3 months (n =578) with all the three direct measurements are represented.

(a) Half-width(a_1)

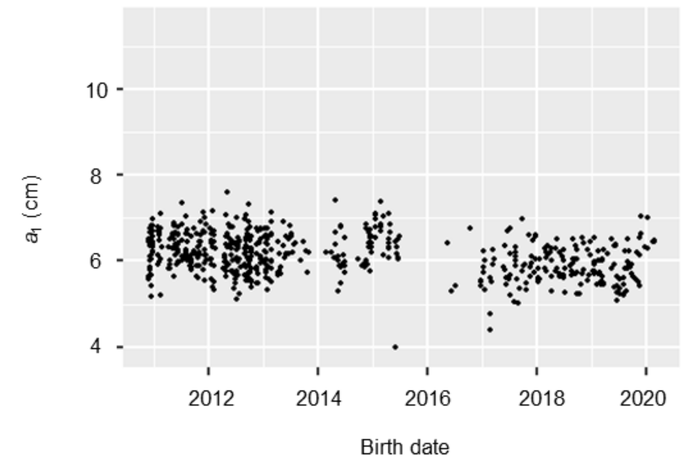

(b) Half-length(a_2)

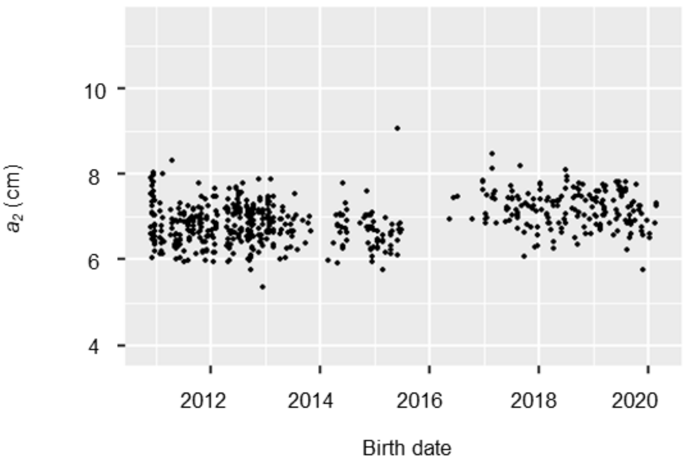

(c) Height(h)

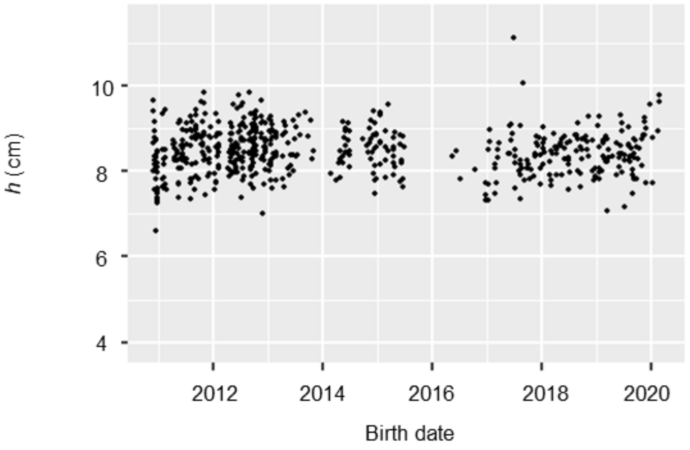

S7 Fig. Scatter plots showing changes in the estimated values for half-width (a_1), half-length (a_2) and height (h) at 3 months of age during 2010-2019.

(a) Half-width (a_1), (b) Half-length (a_2) and (c) Height (h). Infants aged 3 months (n =578) with estimated a_1, a_2 and h measurements derived from the three direct measurements (left tragion-right tragion (LT-RT), glabella-occipital protuberance (G-O), and head circumference (HC)) are represented.

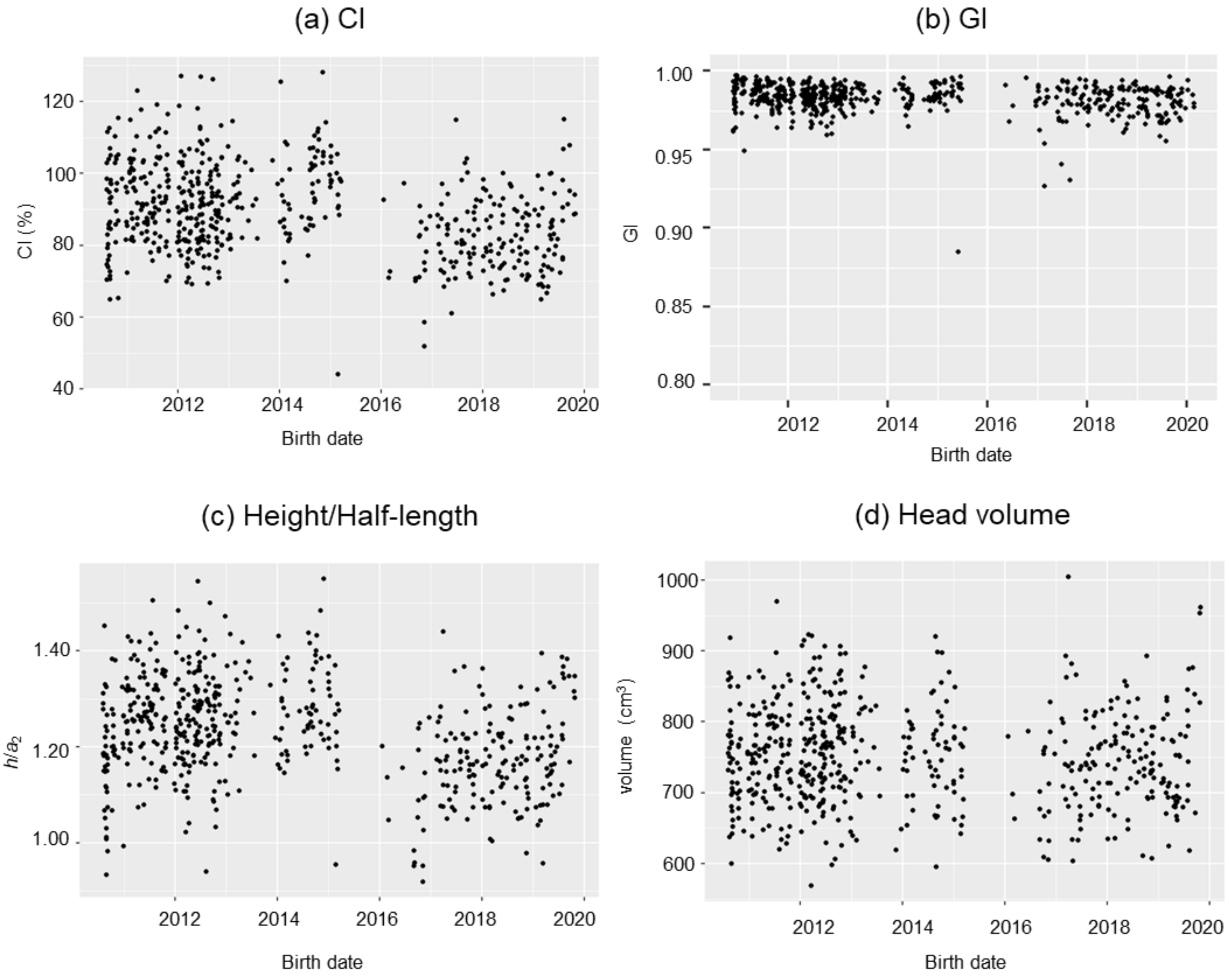

S8 Fig. scatter plots showing changes in the estimated measurements (Cephalic Index (CI), Globularity Index (GI), height/half-length, and head volume) during 2010-2019.

(a)Cephalic Index (CI), (b) Globularity Index (GI), (c) height/half-length, and (d) head volume. Infants aged 3 months (n =578) with CI, GI, height/half-length ratio, and head volume measurements that were obtained from the three direct measurements (left tragion-right tragion (LT-RT), glabella-occipital protuberance (G-O), and head circumference (HC)) are represented.

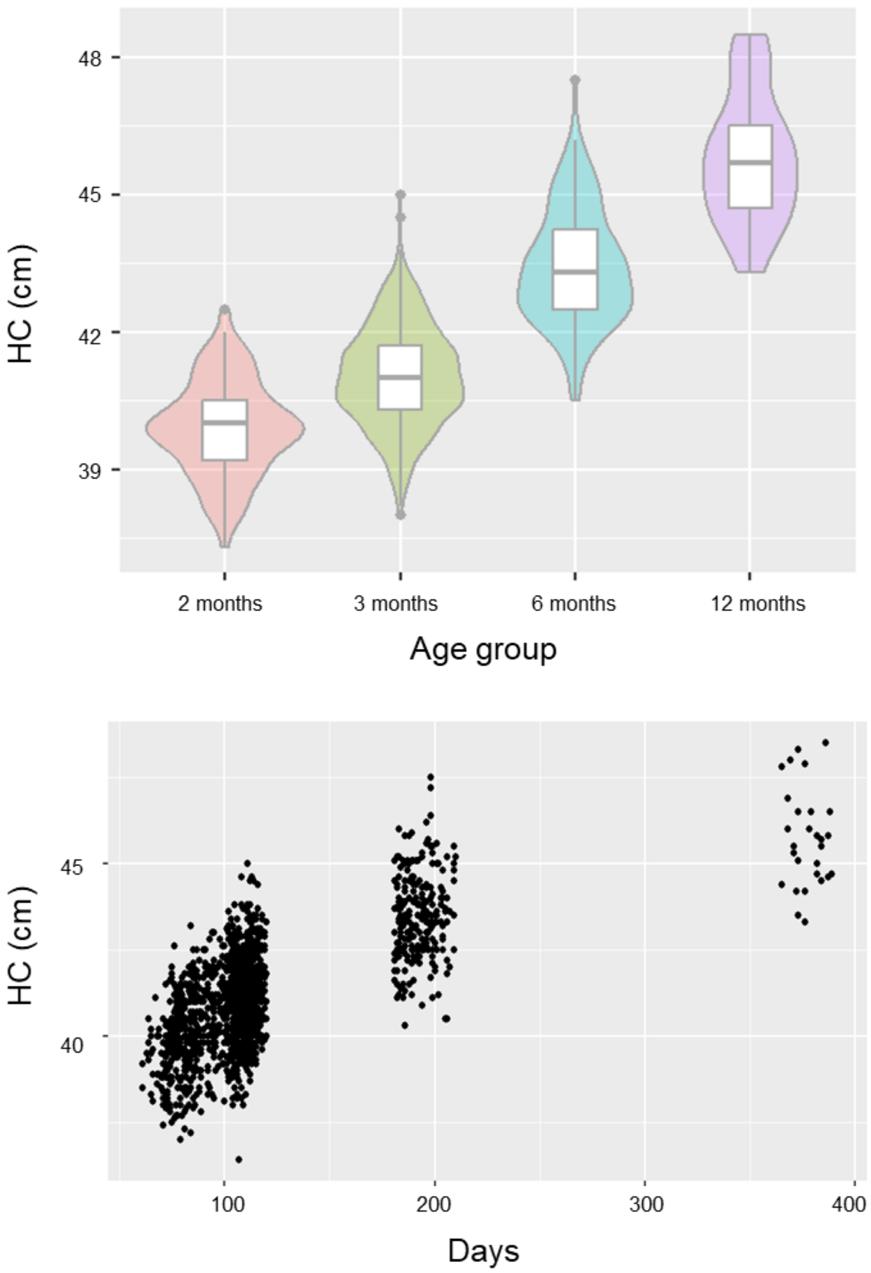

S9 Fig. Boxplots of head circumference (HC) measurement across different age groups and a scatter plot showing changes in the HC during the first 12 months after birth.

The boxplots and the scatter plot represent infants with head circumference (HC) measurements, excluding preterm and age groups other than 2, 3, 6, and 12 months but including those without left tragion-right tragion (LT-RT) and/or glabella-occipital protuberance (G-O) measurements (n = 1901).
