# Supplementary material for: Changes in infant head shape: Developmental trends during the first year of life and secular changes observed in recent years
Source: PLoS One. 2026 Mar 13;21(3):e0344700. doi: 10.1371/journal.pone.0344700 (PMC12987498; doi:10.1371/journal.pone.0344700)
Supplement: S3 File — (PDF) [file pone.0344700.s003.pdf]

Supplementary Scripts

Title

Changes in Infant Head Shape: Developmental Trends During the First Year of Life and Secular Changes Observed in Recent Years

Short title

Developmental and Secular Changes in the Infant Head Shape

Eujin Lee[1], Hama Watanabe[1], Ryoya Saji[2,3], Fumitaka Homae[4,5] Gentaro Taga[1]

[1] Graduate School of Education, The University of Tokyo, Tokyo, Japan

[2] College of Agriculture, Tamagawa University, Tokyo, Japan

[3] Brain Science Institute, Tamagawa University, Tokyo, Japan

[4] Department of Language Sciences, Tokyo Metropolitan University, Tokyo, Japan

[5] Research Center for Language, Brain and Genetics, Tokyo Metropolitan University, Tokyo, Japan

Corresponding author: Eujin Lee

Email: elee715@p.u-tokyo.ac.jp

Session Information

Analyses were performed in R version 4.5.0 (2025-04-11), under Windows11. Key packages used were ggplot2 (3.5.2), car (3.1.3), emmeans (1.11.2.8), data.table (1.17.4).

Example R scripts for comparisons of cephalic index across age groups/birth years, for Fig 3,4,6, and 7.

```
# for conversion of data into tables
library(data.table)
# for calculation of estimated marginal means (EMM), pairwise EMM differences, and effect sizes
library(emmeans)
# for performing Type2 ANOVA
library(car)
#for data visualization
library(ggplot2)
# for color palette
library(RColorBrewer)

# Import data
head_dt <- fread("file_path/head_dt.csv")
# Convert to a data.table
setDT(head_dt)
# Construction of a linear model incorporating cephalic index (CI) as the response variable, with age in months, sex, and birth year as main effects no interactions. For birth year comparisons, replace the model with lm(CI ~ birth_year + sex, data = head_dt).
model <- lm(CI ~ age_in_month + birth_year + sex, data = head_dt)
#Conduct type2 anova
Anova(model, type = "II")
#Calculate estimated marginal means for each age group, adjusting for sex and birth year and 95% confidence intervals using the model's summary output.
emm <- emmeans(model, ~ age_in_month )
summary(emm, level = 0.95)
# Convert the output into a data frame
emm_df <- as.data.frame(emm)
#Performing pairwise comparisons with FDR correction and converting the output into a data frame
pairs_emm <- pairs(emm, adjust = "fdr")
pairs_df <- as.data.frame(pairs_emm)
# Computing effect sizes
```

```
eff <- eff_size(emm, sigma = sigma(model), edf = df.residual(model))
```

#Generating violin and box plots representing raw data distribution with data points and error bars representing estimated marginal means adjusted for sex and birth year, in the below example for CI. For birth year comparisons, replace “age_in_month” with “birth_year”.

```
ggplot() +
      geom_violin(
      data = head_dt,
      aes(x = age_in_month, y = CI, fill = age_in_month), color = "darkgrey",
      trim = TRUE,
      alpha = 0.3) +
   geom_boxplot(data = head_dt, aes(x = age_in_month, y = CI, color = age_in_month), width = 0.25)+
   geom_point(
      data = emm_df,
      aes(x = age_in_month, y = emmean),
      size = 0.5,
      alpha = 0.5
   ) +
   geom_errorbar(
      data = emm_df,
      aes(x = age_in_month, ymin = lower.CL, ymax = upper.CL),
      width = 0.12
   ) + scale_fill_brewer(palette = "Dark2") + scale_color_brewer(palette = "Dark2")
```

<u>Generating the scatter plot of CI vs birth date in Fig 5.</u>

#to recognize birth dates as dates

```
library(lubridate)
head_dt <- mutate(head_dt, Birth_day = ymd(Birth_day))
```

#Generating a plot displaying CI distribution across birth dates.

```
ggplot(head_dt, aes(x = Birth_days, y = CI, color = age_in_months)) +geom_point() +ylim(40,140)
```

<u>Generating the reference merged plot for comparison of the HC data collected in this study with that obtained from the government database in Fig 8.</u>

#lab_data refers to the HC data collected in this study and 1960_data, 1970_data etc., are from the

government database)

```
ggplot()+
geom_point(data = lab_data, aes(x = Days, y = HC), color = "green")+
geom_smooth(data = lab_data, aes(x = Days, y = HC), method = "lm", formula = y ~ log(x), se = FALSE,
color = "black", linewidth = 1.0)+
geom_line(data = 1960_data, aes(x= Days, y = HC), color ="yellow", linewidth = 0.9)+
geom_line(data = 1970_data, aes(x= Days, y = HC), color = "orange", linewidth = 0.9)+
geom_line(data = 1980_data, aes(x= Days, y = HC), color = "blue", linewidth = 0.9)+
geom_line(data = 1990_data, aes(x= Days, y = HC), color = "purple", linewidth = 0.9)+
geom_line(data = 2000_data, aes(x= Days, y = HC), color = "red", linewidth = 0.9)+
geom_line(data = 2010_data, aes(x= Days, y = HC), color = "gray", linewidth = 0.9)+
ylab("Head circumference")+xlab("Age(days)")
```
