# Supplementary material for: Changes in infant head shape: Developmental trends during the first year of life and secular changes observed in recent years
Source: PLoS One. 2026 Mar 13;21(3):e0344700. doi: 10.1371/journal.pone.0344700 (PMC12987498; doi:10.1371/journal.pone.0344700)
Supplement: Attachment — Submitted filename: Response_to_Reviewers.pdf [file pone.0344700.s005.pdf]

Response to Reviewers

ID: PONE-D-25-29078

Title: "Changes in Infant Head Shape: Developmental Trends During the First Year of Life and Secular Changes Observed in Recent Years"

Point-by-point responses to the reviewers' comments:

Journal requirements:

1. When submitting your revision, we need you to address these additional requirements. 2.Please ensure that your manuscript meets PLOS ONE's style requirements, including those for file naming. The PLOS ONE style templates can be found at
   https://journals.plos.org/plosone/s/file?id=wjVg/PLOSOne_formatting_sample_main_body.pdf and
   https://journals.plos.org/plosone/s/file?id=ba62/PLOSOne_formatting_sample_title_authors_affiliations.pdf

Thank you for providing the references. We have reviewed the guidelines and made changes to the font sizes for the subsection titles and table formats accordingly.

2. We note that the grant information you provided in the 'Funding Information' and 'Financial Disclosure' sections do not match. When you resubmit, please ensure that you provide the correct grant numbers for the awards you received for your study in the 'Funding Information' section.

Thank you for raising this point, we will make sure that the "funding information" and "financial disclosure" match when re-submitting.

3. Thank you for stating in your Funding Statement:
   The study was partly supported by Japan Society for Promotion of Science Grants-in-Aid for Scientific Research (23H05425) to G.T.
   Please provide an amended statement that declares *all* the funding or sources of support (whether external or internal to your organization) received during this study, as detailed online in our guide for authors at http://journals.plos.org/plosone/s/submit-now. Please also include the statement "There was no additional external funding received for this study." in your updated Funding Statement. Please include your amended Funding Statement within your cover letter. We will change the online submission form on your behalf.

Thank you for your comment. Regarding the funding statement, we would like to amend as below:
"The study was partly supported by Japan Society for Promotion of Science Grants-in-Aid for Scientific Research (23H05425) to G.T. There was no additional external funding received for this study."

4. Thank you for stating the following in the Acknowledgments Section of your manuscript: We would like to express our thanks to Kayo Asakawa and Yoshiko Koda for data collection; and Keiko Hirano, Tomoko Yoneyama, and Nobue Kanaya for administrative assistance. The study was partly supported by Japan Society for Promotion of Science Grants-in-Aid for Scientific Research (23H05425) to G.T.
   We note that you have provided funding information that is not currently declared in your Funding Statement. However, funding information should not appear in the Acknowledgments section or other areas of your manuscript. We will only publish funding information present in the Funding Statement section of the online submission form. Please remove any funding-related text from the manuscript and let us know how you would like to update your Funding Statement. Currently, your Funding Statement reads as follows:
   The study was partly supported by Japan Society for Promotion of Science Grants-in-Aid for Scientific Research (23H05425) to G.T. Please include your amended statements within your cover letter; we will change the online submission form on your behalf.

Thank you for highlighting this point, we have deleted the funding information from the acknowledgements section in the manuscript.

5. Please upload a new copy of Figure 2, 3, 4, 5, 6, 8, 9 and 10 as the detail is not clear.
   Please follow the link for more information: https://blogs.plos.org/plos/2019/06/looking-good-tips-for-creating-your-plos-figures-graphics/" https://blogs.plos.org/plos/2019/06/looking-good-tips-for-creating-your-plos-figures-graphics/

We apologize for the unclear details. We have uploaded updated images.

6. We note that there is identifying data in the Supporting Information file. Due to the inclusion of these potentially identifying data, we have removed this file from your file inventory. Prior to sharing human research participant data, authors should consult with an ethics committee to ensure data are shared in accordance with participant consent and all applicable local laws.
   Data sharing should never compromise participant privacy. It is therefore not appropriate to publicly share personally identifiable data on human research participants. The following are examples of data that should not be shared:
   -Name, initials, physical address
   -Ages more specific than whole numbers
   -Internet protocol (IP) address
   -Specific dates (birth dates, death dates, examination dates, etc.)
   -Contact information such as phone number or email address
   -Location data
   -ID numbers that seem specific (long numbers, include initials, titled “Hospital ID”) rather than random (small numbers in numerical order)

Data that are not directly identifying may also be inappropriate to share, as in combination they can become identifying. For example, data collected from a small group of participants, vulnerable populations, or private groups should not be shared if they involve indirect identifiers (such as sex, ethnicity, location, etc.) that may risk the identification of study participants.

Additional guidance on preparing raw data for publication can be found in our Data Policy (https://journals.plos.org/plosone/s/data-availability#loc-human-research-participant-data-and-other-sensitive-data) and in the following article: http://www.bmj.com/content/340/bmj.c181.long.

Please remove or anonymize all personal information (<specific identifying information in file to be removed>), ensure that the data shared are in accordance with participant consent, and re-upload a fully anonymized data set. Please note that spreadsheet columns with personal information must be removed and not hidden as all hidden columns will appear in the published file.

Thank you for providing the detailed descriptions of the requirements, we have reviewed them and removed the column for "chronological age(in months)" as it gives information more specific than whole numbers from the data file.

7. If the reviewer comments include a recommendation to cite specific previously published works, please review and evaluate these publications to determine whether they are relevant and should be cited. There is no requirement to cite these works unless the editor has indicated otherwise.

Noted with thank you.

Reviewer1's comments:

1. This manuscript presents a large dataset of manual head measurements in Japanese infants to analyze changes in cranial shape during the first year of life. While the topic is relevant and the sample size robust, the study lacks methodological innovation and relies on simplistic geometric assumptions. The proposed Globularity Index (GI), though conceptually interesting, is not adequately supported by validation or comparison with 3D imaging methods. Figures and tables need improvement, and the discussion fails to explore the clinical or physiological implications of the findings. Overall, the manuscript would require substantial revision to be suitable for publication.

Thank you very much for your overall feedback and clear instructions on which components need improvements and taking the time to raise points that required attention to details.

We agree that the methods used for geometric assumptions were simplistic; however, the current study aimed to develop methods that are simple to allow long-term monitoring as mentioned in detail in our response to comment no.10, and because of the abundance of tape measurement data in the everyday settings. We also agree that GI needs further evaluation, which is mentioned in our limitation section, and

we would like to consider this point in the next step. With regards to the significance of Globularity index, please also refer to our responses to comment no.10.

Relating to the clinical significance, currently no established methods for morphological assessment of the infant head exist other than CI, which lacks the ability to provide details, and the morphological norms are unknown. For this reason, it is challenging to interpret how these findings impact the clinical aspects, but in line 718-735 we mentioned some biological aspects that may be related.

The manuscript has now been revised, with substantial modifications made to the methods and results section. We incorporated a general linear model, or specifically, factorial ANOVA to consider effects exerted by multiple factors, as this point was raised by reviewer 2. Please refer to “Data analysis and visualization” section for further details.

Figures have now been updated for better resolution, and the tables were modified as suggested. Please also see our responses to comment no.4-6.

Thank you again for providing valuable feedback, which allowed us to greatly improve the manuscript.

2. The following points should be addressed: The data used in the study, was already published? If so, please, add link to data and specify the additions of this study to the already available information.

Thank you for your comment. No prior studies employed these data.

3. Table 1 have several mismatches and needs to be corrected or clarified: When talking about age, the sentence “excluding 155 age groups other than 2, 3, 6, and 12 months” is repeated. Please, clarify this as the final number matches the total number of the dataset (1980 infants). Is there really an exclusion? Why? (Other ages could have been included) . If only infants with certain ages are taken into account it should be stated and clarified at the beginning, and not repeated for each table.

Thank you for highlighting this part. We would like to sincerely apologize for the confusion. The submitted data file contained individuals with no information on any of the direct measurements (HC, LTRT, GO) by mistake, and therefore we excluded them from the new data file and recounted the numbers. The numbers in table1 should now match those in the data file.

Regarding the monthly segments, it is included in line 178-182 that the age more concentrated at 2, 3, 6, and 12 months and we have very few for other monthly categories. However, other month categories were still included in the final figure, Fig. 8, the comparative review of HC measurement from this study with those from the government data in the discussion.

Following your suggestion, we have mentioned the exclusion of other monthly categories once at the beginning.

4. In table 1 b the total number of infants is different for birth weight and birth year. There’s no reason to not show

mean and median in age.

Thank you for your comment, in line 150-151 we mentioned that two records were missing for birth year data, therefore it is as intended. We modified the tables and included the median and mean in age in table1(b) following your suggestion.

5. In table 1c total number of infants (sum of all tables) is 914, that does not match previous numbers. I consider that exclusions need to be stated at the beginning, and then, used data needs to be detailed.

Thank you for your comment, as mentioned above in our response to comment no.3, the data file contained those that needed to be excluded. We changed the file and corrected the numbers so that it matches the total of 909.

6. Subsections in table 1 are difficult to understand, I consider the information should be clarified and separated in different tables. Journal submission guidelines specify that subsections in tables should have a consistent number of columns or be divided.

Thank you for your suggestion, we agree that the subsections should be divided and simplified. We modified tables in table 1.

7. Lines 518-532 are not interpretable, a better way to present the information must be found.

Thank you for raising this point, we agree that it is difficult to interpret this section. Instead, we decided to summarize and shorten the descriptions and include tables with details of pairwise comparisons in the supplementary tables for reference.

8. The resolution of all the figures needs to be improved. I cannot evaluate figure 5 as it is not visible.

We would like to apologize for this issue. Please see the updated figures.

9. Explain the upper part of boxplots (Figures 3 and 4).

Thank you for your comment, we would like to apologize for the poor resolution of the figures. Previously the upper part of the box plots displayed significance with asterisks. We now took these asterisks outside the plot area to avoid confusion. Please see the updated figures in better resolution.

10. No discussion is made on the importance of GI and its advantages in comparison with CI. Conclusions or discussion should include the implications of the results.

We are grateful for your constructive feedback. We consider that there are two advantages to GI over CI or existing methods.

1. **Objective 3D information**: GI differs from CI in that it also provides additional objective 3D information, including head height and sphericity. GI will give information on the overall roundness of the head. This 3D information will be useful as physicians likely depend on subjective evaluation such as appearance for assessment of morphological improvements after medical or non-medical interventions, due to lack of established methods other than CI.

2. **Easiness of extracting information and suitability for long-term monitoring:** Additional 3D information can be derived from the readily available tape measurements data using GI. Previous studies with 3D characterization of the head obtained scanning images to visualize the shape rather than estimating from the readily available tape measurements. The easiness of extracting additional 3D information is compatible with longitudinal monitoring required for long-term morphological improvements. We consider that its simplicity is one of the key aspects.

To the best of our knowledge, no studies employed sphericity metrics within the realm of infant development.

We added line 857-864 to mention the significance of evaluating GI, as below:

Line 857-864

"*Yet, extraction of additional objective 3D information including head shape roundness and height from readily available tape measurements should be useful, as in some cases physicians rely on subjective evaluation such as appearance for the assessment of morphological improvements after medical or non-medical interventions, due to lack of well-established methods other than two-dimensional CI. Moreover, the easiness of extracting additional 3D information is compatible with longitudinal monitoring required for long-term morphological improvements.* (7)"

Reviewer2's comments:

1. First of all, I would like to thank the editors for the opportunity to review this manuscript. The study addresses an interesting and relevant topic; however, it requires substantial revisions before it can be considered for publication."

We are grateful for your interest in our research and positive feedback. We made substantial modifications to statistical analyses based on your valuable feedback. Your input helped us improve our manuscript, and we appreciate you taking the time to read including the details.

2. Abstract

   -Authors should specify the design (cross-sectional with repeated measures in a subset), inclusion/exclusion criteria, and the variables used to estimate CI/GI/volume (HC, LT-RT, G-O).

   -Authors should flag upfront the non-independence limitation: records are treated as distinct individuals despite 95 infants with repeated visits.

   -Authors shpuld add n by age group and effect sizes/95% CI for the main results to improve interpretability.

Thank you for your feedback on abstract. We tried our best to incorporate your suggestions as much as possible, to the extent possible within the 300-word-limit.
Regarding the study design, we have added in line 27 that this is a cross-sectional retrospective study. With regards to repeated measures, please refer to our responses to comment no.4. We have included the variable used to estimate CI/GI/volume, and effect sizes for the age group comparisons. However, due to the word limit it was not possible to include effect sizes for birth year differences.

3. Introduction

   -Authors should define more precisely the novelty of the Globularity Index (GI) relative to prior sphericity/compactness metrics and justify why the chosen approximation (ellipsoid surface via Knud Thomsen) is appropriate for infant crania, with references and/or validation.

Thank you for your comment. GI differs from CI (which is currently the only well-established method), in that it also provides additional objective 3D information, including head height and sphericity. While physicians are likely to depend on subjective evaluation such as appearance for assessment of morphological improvements after medical or non-medical interventions due to lack of established methods other than CI, objective 3D information can be derived from the readily available tape measurements data using GI. The easiness of extracting additional 3D information is compatible with longitudinal monitoring required for long-term morphological improvements. Previous studies with 3D characterization of the head obtain scanning images to visualize the shape rather than estimating from the readily available tape measurements. To the best of our knowledge, no studies employed head sphericity metrics within the realm of infant development nor in other healthcare related fields. We added line 857-864 to mention the significance of evaluating GI.
Regarding the ellipsoid estimation, we mentioned in the methods section line 202 that the infant head is generally considered to resemble an ellipsoid as previously reported.

Line 200-202:
"For estimation of CI, head widths, lengths and heights were first deduced from the three direct measurements (HC, G-O, and LT-RT) as shown in ***Fig 1*** *under the assumption that the head resembles an ellipsoid as previously reported (21,22)."*

4. Data and design

   -Authors state that each record was treated as a single infant; however, 95 infants had measurements at multiple visits. This violates independence and likely underestimates the variance. Authors should reanalyze the data using mixed-effects models (with infant identification as a random effect) or, at a minimum, provide a sensitivity analysis that excludes repeated cases.

   -Authors should provide more detail on how HC, LT-RT, and G-O were collected, and define precisely the age bins (30-day windows), including ranges and the distribution per group in the text (not only in tables).

Thank you for raising this point. We consider that infant identification as a random effect should not be a problem as there were only 21 samples which were mostly duplicates among the 909 records with the complete set of three measurements necessary for calculation of CI/GI etc. These 909 records were the data used for all figures except for Fig.8. We mentioned this point in line 176-187 and also added more descriptions about the data composition in the data characteristics section.

Regarding the details of how the measurements were collected, we included the following in the data source section line 125-133:

" *The recorded head shape measurements included the following three: Head circumference (HC) was measured by encircling the head along a horizontal plane passing through the glabella (G) and the occipital (O) protuberance. Left tragion to right tragion (LT–RT) distance was measured along the surface of the scalp, passing through the left tragion, Cz (as defined according to the international 10–20 EEG system), and the right tragion. Glabella-Occipital (G-O) distance was measured along the surface of the scalp, passing through the glabella protuberance (G), Cz, and occipital protuberance (O). All these direct measurements were collected using tape measures.*"

We tried incorporating the details of distribution with age range that were already presented in the modified table1 in the text, but it made it difficult to read. Therefore, we decided to simplify the text and referenced the table instead.

"

5. Geometric approach and assumptions

   -The ellipsoidal model infers a1, a2, and h from HC, LT-RT, and G-O. Authors introduce a factor α = 0.8 to adjust LT-RT per the 10–20 system. This assumption is highly influential yet no sensitivity analysis (e.g., α = 0.75–0.85) nor external validation (a small 3D substudy) is presented. Please add both.

Thank you for your feedback. The factor α = 0.8 was not derived from our assumption but instead reflects the fact that the head circumference was measured at points 10% of the total LT-RT distance away from LT and RT. We added a new reference (ref no.23) as alpha value 0.8 was derived from this. We modified

line 125-133 as above in our response to comment no.4 and 218-221 as below.

Line 218-221

"The over-the-head distance LT-RT was multiplied by α, which was set to 0.8, as the HC line intersects the LT-RT line near T3 and T4 of the international 10-20 system, at points corresponding to 10% of the total LT-RT distance away from LT and RT (23)"

We also performed sensitivity analysis using α = 0.75 and α = 0.85 to see how these differences affect especially CI and GI. As "the HC line intersects the LT-RT line at points near T3 and T4 of the international 10-20 system, corresponding to 10% of the total LT-RT distance away from LT and RT", we defined alpha = 0.8. With respect to the trends observed in CI across age groups, alteration of alpha value did not affect the overall trend that 6 months presented the highest CI, as shown in the below figure.

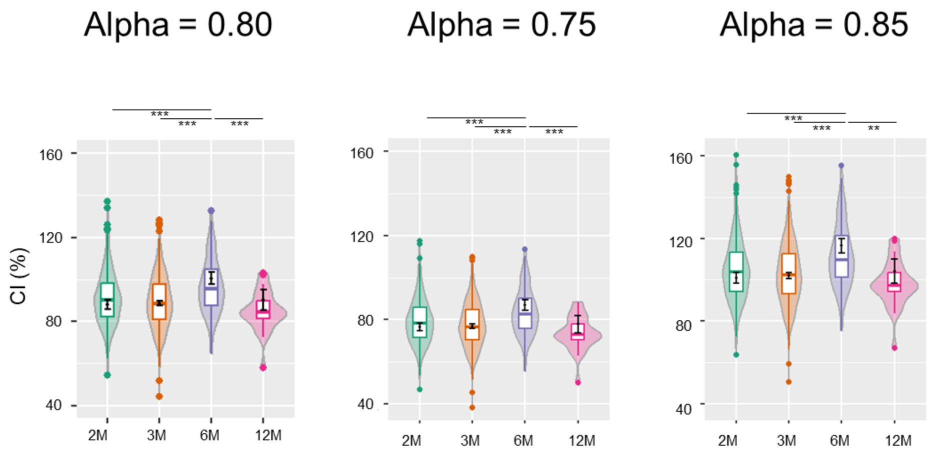

In terms of GI, replacing the alpha value with 0.75 made no difference in the overall trend; the head roundness increasing from 3 to 6 months and decreasing from 6 to12 months. However, changing alpha value to 0.85 made GI decrease from 2 to 6 months, followed by no difference as shown in the figures below. These indicated that although the evaluation remained robust between alpha value 0.75 and 0.8, this robustness did not hold for 0.85. Furthermore, deducing the exact corresponding values for each measurement from the existing studies is challenging as they heavily depend on the reference points and the plane, in addition to differences in age segmentation.

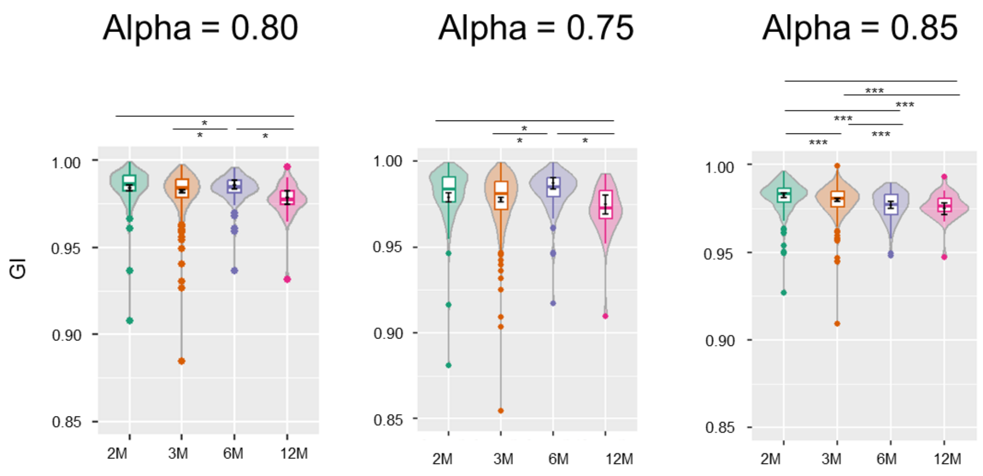

6. GI definition and statistical handling

   -GI is defined as the ratio between the surface of an ellipsoid (Knud Thomsen) and that of a volume-equivalent sphere—geometrically fine.

   -Authors apply Fisher's z transformation to GI "because values cluster near 0.9–1.0." Fisher's z is intended for correlation coefficients; for bounded indices, logit or arcsin-sqrt transforms or beta regression are more appropriate. Please re-analyze GI using an appropriate transformation/model and report whether inferences change.

Thank you for your comment, as suggested we decided to use the raw values and delete transformed GI data.

7. Statistical strategy

   -Authors describe a large battery of tests (Shapiro–Wilk, Bartlett, ANOVA/Welch, Kruskal–Wallis, Nemenyi, Tukey/Games–Howell, Wilcoxon/Fligner-Policello). This multiplies univariate contrasts without global type-I error control. I recommend: Linear/multilevel models for CI/GI/volume with exact age (days), sex, year (and, if available, rater) as covariates; FDR (Benjamini–Hochberg) correction for families of comparisons.

   -Restricting to 3 months may induce bias (exact age within that month, changing composition by year). Please expand with models that include continuous age and test year×age interactions.

Thank you for your thoughtful and insightful feedback. We agree that the statistical approach needs to be revised to consider the effects of multiple predictors and avoid using different sets of univariate tests. Therefore, we changed the method to involve a linear model, or specifically, factorial ANOVA, which is equivalent to generalized linear model with gaussian distribution and identity link. For the pairwise comparisons we applied estimated marginal means, Wald t-test with FDR correction for multiple testing. For the developmental changes during the first year, we would like to stick to age in months as categorical

variables, not exact age in days for several reasons; As the long-term changes are likely to be non-linear and typical patterns are unknown, it may complicate the model and the interpretation. Although we would like to try applying the more advanced models suggested in your comments in the future, in this study, we would like to focus on identifying the differences between age groups than finding the best model. For the developmental changes during the first year, we included age in months, birth year and sex as main effects with no interactions, based on AIC and R^2 metrics.

For the birth year comparisons, we included birth year and sex as main effects with no interactions, and we decided not to include age in exact days, because of the data structure; when the records were subdivided to birth year level, there were biases in the age distribution as well as gaps, with some years concentrating in the middle while others at the two ends of the age range. We considered that it would be risky to draw conclusions for the age (in days) impact and age x birth year interaction, although it was indeed very intriguing and interesting. With the future sets of data, we would like to consider these points, thank you for the great suggestions.

Therefore, for simplicity, we chose these methods. We updated the figures to include violin and box plots depicting the raw data along with estimated marginal means with error bars(CI95%). Please also see data analysis and visualization section for full details.

8. Reproducibility

   -Authors list R packages but provide no code/seed or fully reproducible analysis plan. Please deposit scripts and "session info."

Thank you for your comment.

We have included R script and session information in the supplementary file.

9. Changes with age (2, 3, 6, 12 months) Authors should add effect sizes and 95% CI for pairwise age comparisons, not only p-values.

Thank you for raising this point, we have modified the results to include effect sizes and means+95% CI. Since adding all effect sizes and 95% CI for all combinations of pairs in the text or in the plots can confuse the readers, we would like to include the details in the supplementary information.

10. Sex differences

    -Authors should provide standardized effects (e.g., Cohen's d) and 95% CI to aid interpretation.

Thank you for raising this point, we have modified the results section to move the sex differences to supplementary files and included references to supplementary files in the text for further details for effect sizes and 95% CI.

11. Secular changes (2010–2019; 3 months)

-Authors report antero-posterior elongation in recent years (a1↓, a2↑; CI↓; h/a2↓) with stable volume. This section would be stronger with adjusted models (exact age in days, sex, rater if applicable), multiplicity control, and visualizations showing means + 95% CI per year.

Thank you for your comment, please refer to our responses to comment no.7.

12. Discussion

Authors should incorporate into Limitations the pseudoreplication, the inappropriate use of Fisher's z for GI, and sensitivity to α. Current limitations mention inter/intra-observer variability and the lack of volume validation, but not these issues.

Please refer to our responses to comment no.4-7.

13. Tables and figures

Global assessment: low quality. Substantial improvements in design and content are required to allow unambiguous evaluation of the findings.

Specific actions:

- Readability and formatting: Increase resolution and line thickness; export in vector formats (PDF/SVG); standardize fonts and sizes.
- Axes and units: Label units (e.g., mm) clearly; use consistent scales across all figures.
- Statistical information: Add n per group, means/95% CIs, and effect sizes next to box plots.
- Clarity of the GI: Avoid displaying "GI (z-transformation)" in figures and legends; present the GI on its original scale of 0 to 1 to facilitate clinical interpretation, and if transformation is necessary, show the raw scale and report the transformation only secondarily.

Thank you. We have updated the figures for better resolution and used consistent scales across figures where applicable, for the set of height/a1/a2, and for HC/LTRT/GO. Due to the space limit within the plot, we cannot add n, 95%CI, effect sizes next to boxplots, but instead included sample sizes in the captions and details in the supplementary tables for reference.
For GI, we used the raw values as mentioned above.

14. Key recommendations:

- Independence: Reanalyze using mixed-effects or sensitivity models, excluding repeat visits (identification of the infant as a random effect).
- GI handling: Replace Fisher's z test with logit/arcsine-square root or beta regression; recheck inferences.

Assumption of -α = 0.8: Include a sensitivity analysis and, if possible, external validation.

- Multiplicity/modeling: Reduce reliance on numerous univariate tests; use (multi)variable models with FDR

control.

- Secular trends: Model the year, adjusting for exact age (days), sex, and assessor (if applicable); report the 95% CI.
- Presentation: Improve the quality of tables/figures, add effect sizes/CIs, clarify units and n per group; avoid the "z-transformed GI" in graphs.

We have made substantial modifications to the methods and results sections to incorporate linear models with multiple factors as mentioned in our response to comment 6.
Please refer to our responses to comment no.6 for GI handling, comment no.5 for assumption of α = 0.8, and comment no 7 for secular changes.
