# Supplementary material for: Changes in infant head shape: Developmental trends during the first year of life and secular changes observed in recent years
Source: PLoS One. 2026 Mar 13;21(3):e0344700. doi: 10.1371/journal.pone.0344700 (PMC12987498; doi:10.1371/journal.pone.0344700)
Supplement: Attachment — Submitted filename: Response_to_Reviewers260219.docx [file pone.0344700.s006.docx]

**Response to Reviewers**

**ID:** PONE-D-25-29078

**Title:** "Changes in Infant Head Shape: Developmental Trends During the First Year of Life and Secular Changes Observed in Recent Years"

Point-by-point responses to the reviewers’ comments:

**Journal requirements:**

1. If the reviewer comments include a recommendation to cite specific previously published works, please review and evaluate these publications to determine whether they are relevant and should be cited. There is no requirement to cite these works unless the editor has indicated otherwise.

Thank you for your instructions. We have reviewed the newly suggested publication and decided to include this in the references.

2. Please review your reference list to ensure that it is complete and correct. If you have cited papers that have been retracted, please include the rationale for doing so in the manuscript text, or remove these references and replace them with relevant current references. Any changes to the reference list should be mentioned in the rebuttal letter that accompanies your revised manuscript. If you need to cite a retracted article, indicate the article’s retracted status in the References list and also include a citation and full reference for the retraction notice.

Thank you for your guidance. As mentioned above we have added a new reference and updated the numbers in the list.

**Reviewer 1’s comments**

1. Firstly, I would like to thanks the authors for taking into account most of my comments. I consider that the paper has been greatly improved.

We would like to thank you again for your truly valuable feedback, which has helped us improve the manuscript, especially the discussion section. We sincerely hope that our revisions have addressed your concerns and that the manuscript is now suitable for publication. Thank you very much for your time and consideration.

2. However, there are some further improvements I have to mention:
   -Figures are still low resolution

Thank you for your comment. We have confirmed with the PLOS One editorial team that the figure files meet the requirements. You may be referring to the figures embedded directly inside the compiled pdf file generated by the system. Please click on the link displayed at the top right-hand corner “Click here to access/download;Figure;Figure_X.tif” to see the correct figures.

3. - Figure captions need to be expanded to properly explain what the reader is seeing, some examples:
   • Figures 3 and 4: As I mentioned in my previous comments the symbols at the top are not explained. The explanation is in the response but not in the manuscript.
   • Fig 7. What is government? I assume it is official data but it is not mentioned.

Thank you for your comment. We have mentioned in the captions Fig3 and 4 that the lines indicated the pairing and the asterisks indicated the significance level following your suggestions. We also replaced the word “government” with “national growth survey on preschool children” as it was not clear to the readers where it came from.

4. -The authors mention that no other sphericity metrics are employed in other studies. Some studies exist and should be mentioned and discussion could be improved, for example:
   Grieb, J., Barbero-García, I., & Lerma, J. L. (2022). Spherical harmonics to quantify cranial asymmetry in deformational plagiocephaly. Scientific Reports, 12(1), 167.
   I agree that the method presented in the manuscript has the advantage of “Easiness of extracting information” but it should be discussed by comparison with other methods

Thank you for your insightful feedback. We have reviewed the suggested paper and made references to the method involving spheric harmonics.

We included the comparison in line 867-885 in the manuscript as below:

“*In a recent study, authors applied spherical harmonics to model the distances from the actual head to an ideally fitted ellipsoid using data obtained with a 3D photogrammetric system, based on the assumption that the ideal head shape is an ellipsoid (51). The study investigated the diagnostic performance of spherical harmonics for differentiating infants with deformational plagiocephaly from those without and found that their method outperformed conventional asymmetry indexes.*

*In contrast, the present study characterizes population-level developmental changes in head roundness rather than detecting the deviation from the ideal reference shape. Although in our study GI was derived by dividing the ellipsoid surface area by the volume equivalent sphere surface area, this approach can also be applied to more complex head shapes than an ellipsoid. Since the minimal surface area is achieved by a sphere and deformation of a sphere in any direction, including local surface undulations, leads to an increase in surface area, the GI defined by surface ratios serves as a comprehensive indicator of deviation from sphericity. Using minimal and relatively easy measurements to quantify morphological characteristics has also enabled the collection of data from many infants across a wide range of ages in months. To quantify asymmetry in specific directions or deviations from a typical morphology, more detailed three-dimensional measurements and the establishment of age-related changes in the reference shape will be required.*”
